# Cognition and Interaction: From the Perspective of Daily Therapeutic Landscape of the Coastal Zone

**DOI:** 10.3390/bs13100794

**Published:** 2023-09-25

**Authors:** Shu-Chen Tsai, Hui Wang, Su-Hsin Lee, Zhe Zou

**Affiliations:** 1 College of Arts and Design, Jimei University, Xiamen 361021, China; tsaishuchen@jmu.edu.cn (S.-C.T.); huiwang@jmu.edu.cn (H.W.); 2 Department of Geography, National Taiwan Normal University, Taipei 10644, Taiwan; shlee@ntnu.edu.tw

**Keywords:** pick up, experience, sense of place, assembly, new spatial relationship, human geography

## Abstract

This study explored the relationship between mental and physical therapeutic effects through three dimensions: man–environment relationships, a sense of place, and symbolic landscapes. The study used a combination of qualitative and quantitative research methods. Local residents living in the coastal area of Xinglin Bay were the research objects. Quantitative data analysis revealed that the frequency of residents’ visits was an important variable affecting their physical and treatment perceptions. For those who visit frequently, these visits can evoke memories, which can better express their sensory experience. The text analysis showed that residents picked up two major landscape elements to form the sense of place and symbolic landscape: one is the water body in the coastal zone, and the other is the cultural symbol of the peninsula. Based on untoward event experience, the residents assembled the elements into a new spatial relationship with therapeutic affordance.

## 1. Introduction

Early researchers proposed that the environment can contribute to the health benefits of individuals and society by reducing stress, alleviating anxiety, and reducing feelings of fear [1,2,3]. Even just taking walks in natural environments has been found to be beneficial for health [4].

However, since Geslers [5,6] proposed the study of therapeutic landscapes, it has directed a new topic for the study of medical geography: why are certain places or situations considered therapeutic? There have been a number of studies exploring the healing and health-enhancing dimensions of places [7]. Following Gesler’s work, researchers have recognized the importance of maintaining health and well-being, which extends far beyond healing experiences. “*The themes of therapeutic landscape include man-environment relationships; humanist concepts such as sense of place and symbolic landscapes*” [5]. Moreover, what matters more is the quality of the relationship between therapeutic landscapes and the individual’s experience [7]. This shift acknowledges that places inherently do not possess healing properties; instead, it is through the dynamic interaction between individuals and their environment that opportunities for health and well-being are generated [8].

In recent years, there has been significant progress in the research on therapeutic landscapes within the physical, social, and symbolic dimensions, particularly regarding “nature-based” therapeutic encounters [7]. Notably, there has been fruitful exploration of sensory responses to the environment, with a focus on engaging with the processes and temporalities of intimate, visceral place sensing [9]. “Nature-based” therapeutic encounters view the body as an instrument for perceiving and sensing the social environment, rather than merely as a container. Rather, bodies help constitute that reality, through our doings within it [10]. We must delve into a more intricate understanding that acknowledges the significance of our bodily experiences in the world. It involves comprehending how we actively create and transform the world through skillful sensory activities [11]. On the other hand, an alternative perspective suggests that external bodily injuries can stabilize when participants become aware of bodily changes, recognize the impact of external materials, and engage in reciprocal changes within the body [12]. Therefore, bodily health should not be seen merely as an individual quality but rather as emerging from attunements and resonances between bodies and materials [13].

Currently, research on environmental healing tends to focus on green landscapes (for example, parks, green spaces, forests, mountains, and even virtual green spaces [14,15,16,17]), and quantitative indicators are predominantly used. This approach can be considered a “symptom-oriented” method, where specific themed healing environments such as hot springs and forests (distinct from everyday living spaces) provide possibilities for users to temporarily escape emotionally stimulating situations [18,19]. While these environments can achieve immediate physiological stress reduction (as measured by indicators) [14,19,20,21,22,23], the long-term healing effects remain unknown, as most studies only involve one [14,15,22,23]. Although significant, the effects were observed only in the short term, highlighting the limitation of capturing cross-sectional data and virtual experiences [15]. It is important to discuss the possibility that participants may experience short-term negative emotional displacement due to the enchanting scenery, and this should be considered from different perspectives.

Cognitive control is the ability to process information over time to guide behavior in accordance with current goals [24]. Recent research has also found that cognitive control is a key feature in adapting our behavior to environmental and internal demands [25]. Research indicates that as the body is in constant contact with the environment, more cognitive behavior emerges from situational action [26]. Previous research has also found that both landscape restoration and psychological restoration can be recognized simultaneously in body–environment interactions [27]. This study will further deepen the research on the relationship between interactive behavior and therapeutic landscape.

Despite data indicating the therapeutic nature of places such as forests, mountains, or hot springs in terms of participating in healing or enhancing health, there has been relatively less consideration of the dynamic relationships that underlie these therapeutic effects [7]. What is more, the proposal of a therapeutic landscape stems from the resistance to the hegemony of the biomedical model [6]. We should focus on the interaction between people and the environment, rather than chasing which environment has a healing function. The value of living in the present is deliberately excluded in various studies that pursue amazing experiences. To reproduce this value is the main purpose of this study.

### 1.1. The Inseparability of the Body and Everyday Living Space

Research suggests that long-term and immersive interactions between individuals and their environment are better able to meet diverse therapeutic needs [28]. Well-being is a state beyond the individual body index and also comes from the positive development between the body and the surrounding environment [13]. Therefore, this study responds to scholars who argue that the body should be repositioned within a broader interdisciplinary discourse surrounding nature, health, and well-being [9,10]. Whether the subject matter of traditional landscape ideas is derived from cultural geography, human geography, structuralist geography, and holistic health principles, the internal meaning implicit in everyday activities is one important reason for the therapeutic efficacy of landscapes [6].

Bachelard gives primacy to deep or archetypal emotions that come not from the subject but from living space [29]. It is everyday recreation that takes them out of their subjective and human-centered emotional states into the spatial-temporal depths of the relational state of “well-being” [30]. Even in just a small corner of the world, focusing on the connection between emotions and living space can foster a more expansive worldview [31].

### 1.2. The Therapeutic Imagery of the Coastal Zone

The cultural interpretation of the coast is constantly evolving, from its enjoyment amongst the Ancient Greeks and Romans as a place of pleasure and beauty [28]. And then, the ocean is also filled with fear. The sea has been associated with various metaphors of rage and symbolizes various wild and untamed creatures. “*The ocean dances with a mane of lions; the sea spray is likened to “the drool of sea monsters” and is said to cling to their claws*” [29].

Human geographers have focused more precisely on the coastal zone. The characteristics of concave coasts evoke a sense of safety, while the expansive horizon stimulates human adventurous desires. Especially throughout the 19th century, the coastal zone provided happiness and health to humans, with its value surpassing its economic output [32]. It was only after people recognized the health benefits of sea bathing that health enthusiasts turned their attention to the coast, shifting from thermal springs [33,34]. The coastal zone has also served as an environment for sustenance, learning, and the earliest human habitats [35]. Cultural geographers [31] have commented on the potential of the coast to “*generate a palpable intensity of feeling*” [36].

Research has shown the therapeutic benefits of coastal restoration [37], including physiological, mental-emotional, and creativity-related benefits [38,39,40]. The coastal zone is also regarded as a daily therapeutic space [28], where the frequency of visiting blue spaces is positively correlated with psychological well-being, happiness, and physical activity levels [41]. Moreover, interactions with the coast often involve enduring connections [28]. Studies have also shown that emotional attachments formed in everyday life in the coastal zone can lead to a profound understanding of the interests at stake in local development and inspire actions to play a role [42].

Since the 19th century, the coastal zone has seemingly become synonymous with holidays and leisure, leading to the neglect of its significance as a daily living space for local residents. Therefore, the therapeutic landscape of the coastal zone is lacking in previous studies to separate the recreational components. Furthermore, compared to green spaces such as mountains, forests, parks, and gardens, the therapeutic landscape of the coastal zone has received less attention from the academic community [28]. However, within the broader literature, the coast has been conceptualized as a “therapeutic landscape” [8,9,43].

So, which kinds of elements of everyday life in the coastal zone come together to form the therapeutic landscape? How and why? This study attempts to address this absence through engagement with two related bodies of work: the relationship between daily life and body. Moreover, the process should be a state of long-term interaction between people and the coastal zone, focusing on the sense and memory of previous experience.

### 1.3. Sense and Memory of Previous Experience

People experience the world in terms of how they can act—in terms of their effectivities or action capability [44]. A percept may be considered to be the mental image of the external environment, and it is clearly based on two kinds of information: input from the senses and memory of previous experience [45]. Following the ecological viewpoint that human beings seek survival in the environment, for Gibson [46], senses adapt to the environment due to continuous evolution, and the sensory nervous system picks up information and forms perception. Meanwhile, invisibility, adjusting sensory input, selective attention, and other perceptual activities can make information an effective stimulus and create environmental affordances [46].

Therefore, the key point is how to pick up senses and memories of previous experience in people’s daily life activities in coastal areas. According to the word definition of sense, sense is an ability to understand, recognize, value, or react to something, especially any of the five physical abilities to see, hear, smell, taste, and feel [47]. However, only kinaesthesia (muscle movement) perception, vision, and touch can make human beings have a strong sense of space and space quality, and movements are the basis of the awareness of space [48]. It is pointed out that primates can improve the quality of perception through the extension and refinement of motor investigation, and the evolution of vision is an important basis for animal evolution. Animals can understand the surrounding environment well so that they can control the environment for their own direct interests; visual, somatic sensory, and auditory inputs will be analyzed and integrated with the motor and proprioceptive patterns [45].

People’s feelings are not formed by individuals but by long-term memories and expected results of many experiences. “*in English, “I see”, means “I understand”*” [48]. Seeing has a kind of recogninized, which includes a selective and creative process. Taste activities are related to touch and smell. For example, licking a candy will find the shape of the candy and the taste of caramel. From the previous examples, it can be inferred that people use their senses to send complex commands to the life world [48]. Therefore, visit frequency is the intersection of space and behavior, and it is also one of the methods to continuously filter environmental information; on the one hand, it is selected, and on the other hand, it is constructed.

Memory is very important, as it is not only an experience record of an event but also a generalization of spatial relations. We can get the information in the environment from the results of manipulating objects, and generalization can deepen and extend these spatial relations indefinitely [45]. The time factor can exert a greater generalization effect. It is pointed out that the environment under the influence of time is not just a three-dimensional pattern, but primates can perceive it as an “*assemblage of objects*” [45].

The perceptual process formed by the interaction between people and the environment is the core of environmental behavior. The environment provides information to stimulate the senses, and perception is the result of people’s filtering of environmental information [49]. The course of perception cannot separate people from the environment because what people do in the environment determines perception [50].

The research on coastal communities points out that public participation can be an effective way to deal with environmental issues; the degree of participation is an important indicator, and frequency is usually the primary factor [51,52]. However, there is more information behind the frequency, such as residence time and activity type, which can obtain the interaction between people and the environment [53].

Tourists are different from residents. The characteristic of tourists is to constantly seek new places, and in the new environment, they will lack some sensory support. Therefore, they always think that “*vacation areas, however delightful, seem unreal after a time*” [48]. Therefore, in the study of cultural tourism, the frequency of use is regarded as the primary factor to verify the authenticity [54]. In contrast, the awareness of local residents is very important to the decision making of surrounding environment development, and only the participation of residents can make sustainable development possible [52]. People know the neighborhood very well, and even if the frequency of visits is low, it is possible to consciously and theoretically gather places into a spatial relationship [48].

On the other hand, Gesler notes that “*what is therapeutic must be seen in the context of social and economic conditions and changes*” [55]. Durling period of the COVID-19 pandemic, which occurred between 2020 and 2022, has indeed brought about changes in the relationship between individuals and spaces. The experience of lockdown has made people more aware of their connection with nature [56,57]. Studies on lockdown during the pandemic have indicated that the absence of interactions with the coastal zone can disrupt the imagery of home [58].

“Place is a special kind of object. It is a concretion of value, though not a valued thing that can be handled or carried about easily; it is an object in which one can dwell” [48]. The imagery of home serves as a sanctuary, a place of healing, regardless of one’s physical location. However, understanding how individuals establish a sense of home and emotional connection with the coastal zone is a crucial aspect to consider.

Whether it is abstract or concrete symbols, it is the significance for human beings to use it to express meaning [5], as cultural values, social behavior, and individual actions worked upon particular localities over a span of time [59]. The cultural landscape can be viewed as a product of symbolic action; it reveals structures or represents cultural images [60,61,62].

There is too much of the accepted pattern of daily living to warrant reflective thought. In particular, untoward events will force us to reflect [48]. For example, the aforementioned COVID-19 virus infection, unpredictable risks, and troublesome experiences often lead to physical and mental troubles, such as anxiety, depression, memory loss, or stress [63,64,65,66,67,68]. Such special experiences will lead to reflection [69,70,71,72], event memory [73,74], and behavioral changes [75,76].

**Figure 1:**
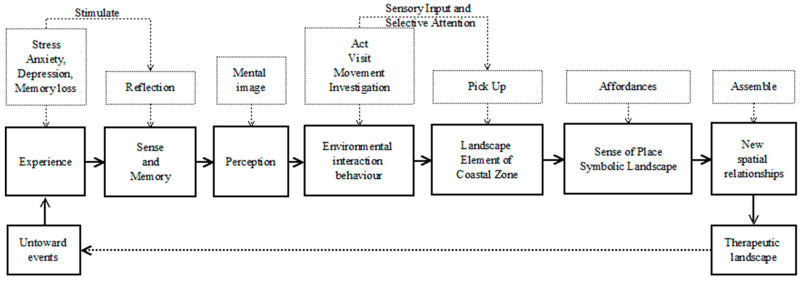
The concept of this study.

Human geographers and psychologists pay great attention to the interaction between people and the environment. No matter whether the words used are movements, acts, visits, or investigations, they are all seeking to adapt to the environment. The behavior change formed by reflection and memory from special experiences is characterized by the interaction between people and the environment. Under the stimulation of environmental behavior, people consciously pick up the landscape elements in the coastal zone to form a sense of place and a symbolic landscape with affordances; after a new spatial relationship is assembled, it becomes a therapeutic landscape and comes back to heal the wounds caused by the untoward event (Figure 1).

## 2. Materials and Methods

This study is a continuation of the landscape resilience perspective [27], with a specific focus on the relationship between local residents and their everyday living spaces. In addition to utilizing literature and oral data as suggested by Geslers [6], scholars have also recommended the careful use of interviews, combined with more structured techniques, to record the forms and characteristics of individual–environment interactions, leading to a deeper understanding of the interactive relationship between people and their environment [7]. Therefore, this study further adopts a mixed-method approach, combining qualitative and quantitative research methods, to explore therapeutic issues and provide a more profound response to the people-centered nature of social science.

This study adopts a qualitative research approach to explore the methodology of dynamic processes. It posits that the more frequently the body is used in the environment, the more cognition emerges from the dynamic activities of contextual actions, leading to an awareness of interactive relationships, a sense of place, and symbolic landscapes. The research methodology involves the use of participant observation and in-depth interviews to collect data. After data categorization and logging, SPSS is employed for data analysis.

### 2.1. The Study Area

There has been a substantial increase in recent years in scholarship exploring the therapeutic landscape experiences in China [9]. These studies are predominantly located in the northwest region, exploring yellow sand therapy [43,77] and targeting the elderly population within urban areas [78,79]. However, there is a notable lack of research on therapeutic landscapes in coastal areas. Therefore, this study sets its research location in the fastest-growing city along the southeast coast.

Prior to 1980, China’s focus on resource utilization was primarily centered around land resources, with relatively less attention given to marine resources [80]. However, with rapid economic development, coastal provinces in China have become the fastest-growing regions. The high intensity of coastal land development and dense population concentration, coupled with investments from coastal cities and foreign countries, have intensified maritime economic activities. These intensive marine utilization activities have led to significant social and environmental changes, increased resource and environmental pressures, coastal pollution, and a decline in ecosystem health, resulting in escalating potential environmental risks [81,82,83,84]. Xiamen City, as early as 1994, became a pioneer in coastal management in China and a pilot city for the Partnerships in Environmental Management for the Seas of East Asia (PEMSEA) organization [85]. Therefore, coastal governance in Xiamen bears the responsibility of the sustainable development of marine resources and has received strong political support [84]. The development goals have been oriented toward a tourism city. From 2010 to 2021, the Xiamen municipal government shifted the function of the coastal area from tidal flat aquaculture to tourism and recreation, thereby transforming the entire coastal landscape [84].

**Figure 2:**
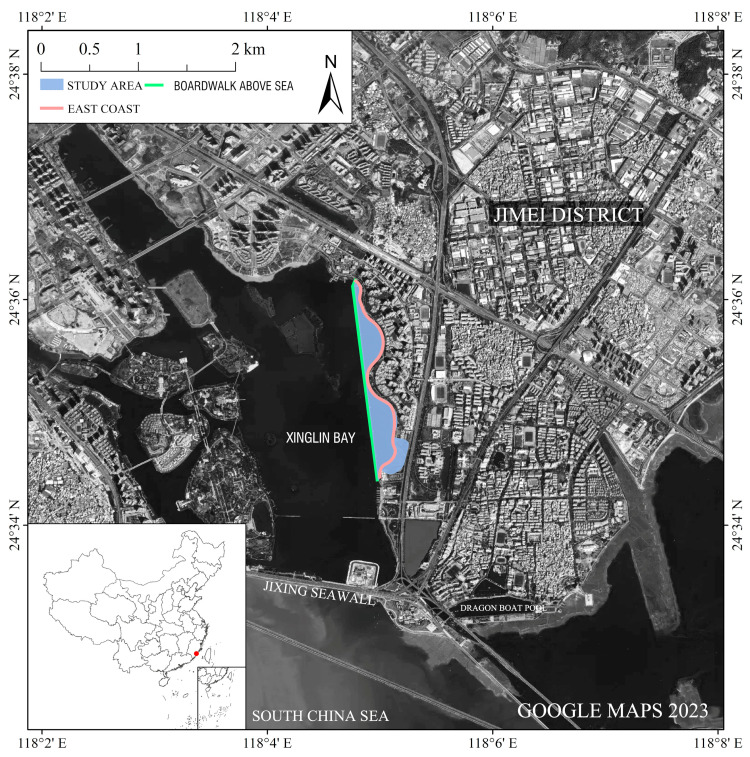
Study area.

Xinglin Bay is located in the center of the Jimei Cultural and Educational District of Jimei Peninsula, attracting a large population due to its educational resources [86]. It serves as a daily living space for Xiamen local residents. In 1979, the formation of a semi-enclosed water area occurred due to the construction of beach embankments, resulting in a unique characteristic of “half seawater, half freshwater” in the area. However, the rapid deterioration of the ecological environment took place as a result of industrial wastewater being discharged into Xinglin Bay from upstream industrial developments. Subsequently, Xiamen City actively promoted coastal tourism and placed emphasis on the scenic benefits of the natural environment, leading to efforts to restore the ecological landscape. During the restoration of the Xinglin Bay landscape belt, a strategy of natural succession as the primary approach, supplemented by human management was adopted [87]. This study focuses on the eastern coastal area of Xinglin Bay, including the boardwalk above the sea (for cycling and jogging), and the landscape belt on the east coast (with large grasslands, trails, water revetments, etc.), which spans a length of 2.6 km (Figure 2).

### 2.2. The Study Content

The study selected local residents of Xiamen City as the research subjects from all interview samples. Through in-depth interviews, information such as the age of the interviewees, frequency of visits, purpose of visits, interactive behaviors with the environment, and specific physical and mental responses were collected.

The first stage of the study was interviews. The content contains a total of six questions. The first three questions are mainly designed to collect objective information, including age, identity, and frequency:

1. What is your age?
2. You’re a nearby resident or a tourist?
3. How often do you visit here per week?

The next three questions focus on the interviewee’s behavioral types, physical and mental changes, and previous experiences. Open-ended questions allow the interviewee to answer without restrictions. As long as the answer content involves two sets of keywords, the interviewer will go deeper into the interview. The theme of the first set of key-words is the sense of place, such as feelings, memory, introspection, experience, etc. The theme of the second group of keywords is symbolic landscape, for example, features, characteristics, symbols, security, and stability:

4. What is the main purpose of your visit?
5. How did you feel physically or mentally during your stay (rowing, running, jogging, walking, cycling…) here?
6. What element/thing attracts you most here?

The second stage is to convert text into category data and establish coding standards. The independent variables are gender, frequency, status, and age. The dependent variables are purpose, physical and mental feelings, and environmental interaction. A rating scale was developed and variables were picked up from the interview texts for coding.

“Behavior of environmental interaction” is divided into three categories:

1. “sensory” (involving the five physical abilities of sight, hearing, smell, taste, and feeling).
2. “body” (movement and fitness).
3. “both” (both of the above categories are involved).

We logged the audio recordings of the interviews as verbatim transcripts. Text material was then manually coded and categorized.

“Perception of therapeutic type” is also divided into three categories:

1. “Therapeutic Landscape”: perceived improvement in the environment, such as changes in water/air quality or migratory birds (landscape); expressions related to exercise and fitness, such as health.
2. “Psychotherapy”: There is a tendency to improve the mind. For example, the text contains content that makes the mood happy, such as feeling relaxed (or releasing stress).
3. “Both” (both of the above categories are involved).

Each category is assigned a number corresponding to the quantitative analysis. According to the third question, the respondents’ visit purposes can be classified into four categories: health, relaxation, scenery, and stress. The operational definitions of the above four categories are as follows: Health: expressions related to sports and fitness. Relaxation: making one feel happy. Scenery: a combination of natural or artificial elements in the environment. Stress: overload caused by life or work (Table S1).

After a two-week participatory observation, it was found that most visitors to the coastal area were local residents. Therefore, it was decided to select the time periods with the highest population density and conduct in-depth interviews with people present at the site. Ultimately, the study conducted interviews during the following time periods: 12–16 October 2022 and 8–9 April 2023, from 7:00 to 9:00 a.m. and 4:00 to 7:00 p.m. The interview locations were the boardwalk above the sea and the eastern landscape belt. A total of 97 people were interviewed, with 89.7% of the total sample consisting of local residents, amounting to 87 individuals.

The interviewees from the boardwalk were categorized as Sample A, while those from the coastal area were categorized as Sample B (Table S1). All interviewees gave their informed consent for inclusion before they participated in the study. In accordance with research ethics, the personal information of the interviewees is protected, and the interviewees are represented by codes.

### 2.3. Data Processing

The data obtained from interviews were categorized and classified in Excel according to interview time, interview location, gender, visit frequency, identity, age, behavior, visit purpose, perception type, and physical sensations. This process transformed the textual data into categorical data.

The data were then subjected to statistical analysis using contingency tables and chi-square tests to explore the relationships between different variables. The research hypothesis assumed that the two variables in the contingency table were independent, and this assumption was tested using the chi-square test. If the chi-square value is significant and the corresponding *p*-value is less than 0.05, the null hypothesis is rejected, indicating a correlation between the two variables. Conversely, if the *p*-value is greater than 0.05, the variables are considered independent. In the calculation of the chi-square test, it is generally recommended to have more than 80% of the expected cell frequencies greater than five to avoid inflated chi-square test results.

Under the aforementioned conditions, a larger value of X^2^(1) indicates a stronger correlation between the two variables [88]. IBM SPSS Statistics 26 was used to analyze the associations between visit frequency, gender, environmental interactive behavior, and healing perception. To conduct a more precise chi-square test, the visit frequency variable was further consolidated into two categories: “Seldom” and “Visit Every week”.

$$x^{2}=\sum_{i=1}^{k}\frac{{\left(f_{i}-np_{i}\right)}^{2}}{np_{i}}$$

As for the analysis of environmental elements of sense of place and symbolic landscape, keywords from the verbatim transcripts of interviews were extracted through text analysis. Sense of place is a keyword that satisfies biological needs, including food, water, reproduction, rest, dwell, home, memory, etc. The environmental elements of symbolic landscapes are represented by keywords related to concrete or abstract things that can represent cultural values, social behavior, and individual actions of geographical features.

## 3. Results

### 3.1. The Age, Frequency, and Purpose of the Interviewees

**Figure 3:**
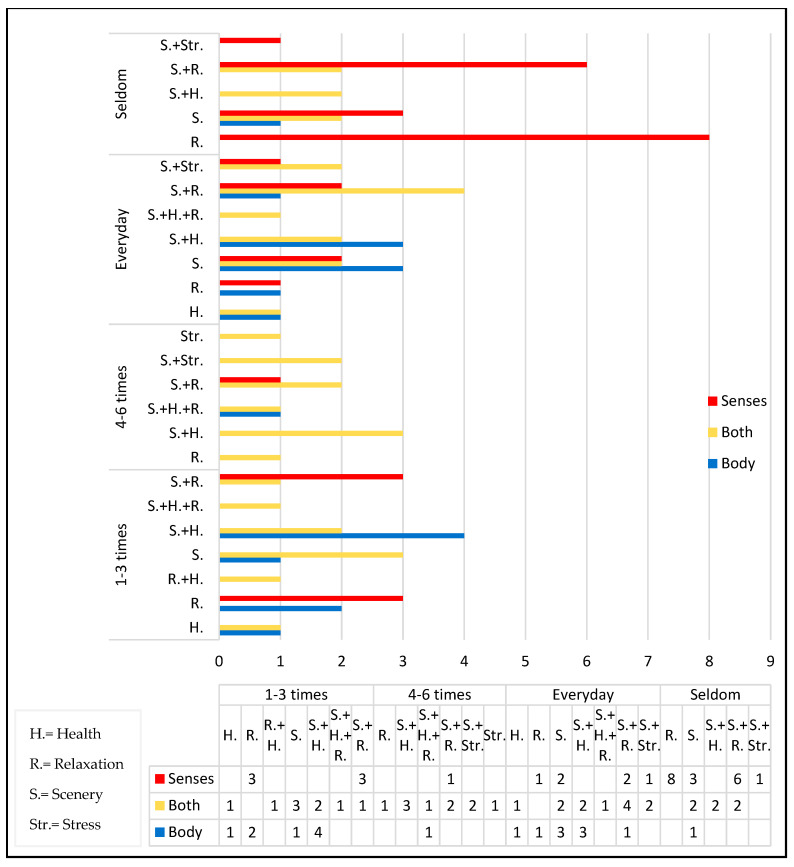
Statistics on age, frequency, and purpose.

A total of 87 samples living in Xiamen were selected in this study. Among them, there were 37 males and 50 females, accounting for 42.6% and 57.4% of the total number of interviewees, respectively. According to the statistical results (Figure 3), the data on age, frequency, and purpose are described as follows:

The age range was divided into five categories: “Under 20”, “20–35”, “36–45”, “46–65”, and “Over 66”. The “20–35” age group accounted for the largest proportion at 35.6%, followed by the “46–56” age group at 29%. The age groups “Under 20” and “Over 66” had the fewest visits, accounting for 4.6% and 5.7%, respectively, with a combined percentage of 10.3%.

The visit frequency is categorized into four groups: “Seldom”, “1–3 times/week”, “4–6 times/week”, and “Everyday”. Among these categories, 71.2% of the total interviewees visited the coastal zone on a weekly basis. The “Seldom” visitors accounted for 28.7% of the total interviewees. The number of interviewees visiting “Everyday” was 27, which represents 31% of the total. The categories “1–3 times/week” and “4–6 times/week” accounted for 26.4% and 13.8% of the total, respectively.

The purpose of the visitors is diverse, and from the bar graph, it can be observed that the scenery is the main factor attracting people to visit. Other factors include stress, scenery, and health, all of which are reasons for visiting the coastal zone. Among them, the combination of “scenery and relaxation” has the highest proportion, accounting for 25.29% of the total interviewees, reflecting the need for people to relax both mentally and physically in the coastal zone. The proportion of visitors coming for “health” reasons accounts for 36.79% of the total interviewees (including stress factors).

### 3.2. Analysis of Body–Environment Interaction Behaviors

#### 3.2.1. Visitation Frequency as a Factor Influencing Environmental Interaction

The cross-analysis table of visitation frequency and behaviors shows that the frequency of “Seldom” visits is 24, accounting for 27.6% of the total, while the frequency of “Visit Every week” visits is 63, accounting for 72.4%. The behavioral engagement of the “Visit Every week” visitors is significantly higher compared to the “Seldom” visitors. Within the “Seldom” frequency range, 17 individuals engage in sensory and environmental interactions, accounting for 70.8% of the total within the “Seldom” frequency category. Among the “Visit Every week” frequency group, 31 individuals simultaneously engage in sensory and body–environment interactions, representing 49.2% of the total “Visit Every week” frequency, nearly half of the population.

Additionally, 28.5% of individuals directly engage in body–environment interactions, indicating that the high frequency of visits and the significant interaction between their bodies and the environment suggest that the coastal zone of Xinglin Bay has become an integral part of their lives.

The results indicate that there is a significant difference between visitation frequency and environmental interaction behavior, demonstrating a strong association between the two, χ^2^(2, N = 87) = 18.066, *p* = 0 (<0.05), *Phi* = 0.46 (Table 1).

Those who visit less frequently use few sensory and physical functions, and some samples mainly use vision and can express fewer feelings (please refer to Table S1 for numbers and contents). For example,

“*The scenery is quite pleasing*.”

(A-2)

“*Appreciating the sunset*.”

(A-7)

“*Contemplating the scenery*.”

(A-9)

“*I haven’t noticed any significant changes*.”

(A-39)

“*Taking a look at the scenery*.”

(A-56)

“*Due to proximity, I decided to take a leisurely stroll in this vicinity*.”

(B-25)

Although there are samples with low frequency, the function of a relaxing mood can be achieved only by walking, but it lacks the experience of senses and memory.

“*Taking a walk and enjoying the scenery, the environment here is comparatively pleasant*.”

(A-50)

“*strolling has improved my physical and mental well-being. It’s a common sight to see people fishing in the area*.”

(A-51)

“*The environment here is relaxing, and the overall surroundings are pleasant*.”

(A-53)

In contrast, those who visit frequently can recall memories and tell the earlier time–space relationship (A-13; A-22; A-29; A-36; A-44; B-4; B-7; B-10; B-12; B-13; B-19; B-24; B-34). These samples can use adjectives of time: *During my childhood* (A-13); *more than half a year* (A-22); *During autumn* (A-42); *After working here for over 20 years* (A-44); *During autumn and winter* (A-47); *During the autumn and winter seasons* (B-1); *Due to the pandemic restrictions* (B-4); *During spring (March to May)* (B-7); *a couple of years ago* (B-10); *A few years ago* (B-12); when *I served as a soldier here in 1987* (B-19); *Since 2006–2007* (B-19).

Moreover, those who visit frequently have more ways of environmental interaction, which can better express their sensory experience:

“*childhood memories and to perceive the passage of time*.”

(A-13)

“*recently, there has been an increase in the number of people, which has unfortunately led to more littering*. *The grassy areas are quite lush, and now it has become livelier with many organized camping activities. After picnics, there tends to be a significant amount of trash left behind*.”

(B-21)

#### 3.2.2. Gender Does Not Influence Spatial Choices in the Coastal Zone

In this study, the sample size of females is higher than that of males. Therefore, the aim is to test whether gender influences the differences in perceiving the coastal zone as a daily therapeutic space.

The statistical analysis results of gender and environmental interaction behavior indicate no significant differences, χ^2^(2, N = 87) = 3.671, *p* = 0.160 (*p* > 0.05), *Phi* = 0.20. Therefore, gender does not influence the preference for the coastal zone as a daily therapeutic space.

In the female group, the number of individuals engaging in sensory and environmental interactions is 22, the number of individuals engaging in body–environment interactions is 9, and the number of individuals engaging in both sensory and body–environment interactions is 19. These numbers represent 44%, 18%, and 38% of the total female interviewees, respectively (Table 2).

In the male group, the number of individuals engaging in sensory and environmental interactions is 9, the number of individuals engaging in body–environment interactions is 10, and the number of individuals engaging in both sensory and body–environment interactions is 18. These numbers represent 24.3%, 27%, and 48.6% of the total male interviewees, respectively.

These results indicate that although the proportion of female visitors to the coastal zone is higher, the proportion of males engaging in body–environment interactions is higher than that of females.

Most studies have not found the influence of gender differences on the overall cognitive ability [89]. Some studies have found that men and women use different orientations to understand the environment [90], which is consistent with the statistical results of this study.

#### 3.2.3. Differences Exist in the Frequency and Interaction with the Environment Based on Gender

In order to further investigate the interaction behaviors between different genders and the environment, it is necessary to examine whether differences arise due to variations in visitation frequency and to further analyze the data.

**Figure 4:**
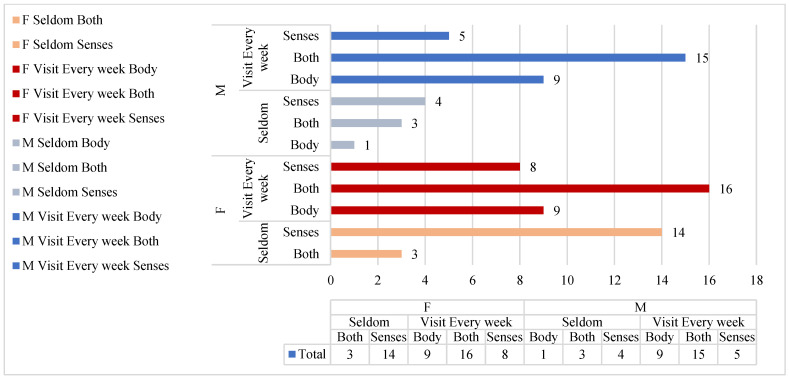
Comparing differences between gender, frequency, and interaction with the environment.

The results indicate that within the female group, in the “Seldom” visitation frequency, there were 18 individuals utilizing “Senses” interaction and 4 individuals utilizing “Both” interactions. In the “Visit Every week” frequency, there were 9 individuals utilizing “Senses” interaction, 9 individuals utilizing “Body” interaction, and 16 individuals utilizing “Both” interactions. On the other hand, the visitation frequency distribution among males was relatively even. In the “Seldom” visitation frequency, there were five individuals utilizing “Senses” interaction, three individuals utilizing “Body” interaction, and four individuals utilizing “Both” interactions. In the “Visit Every week” frequency, there were 5 individuals utilizing “Senses” interaction, 9 individuals utilizing “Body” interaction, and 15 individuals utilizing “Both” interaction (Figure 4).

The results show that there is a highly significant difference between the visitation frequency and environmental interaction behavior among females, indicating a strong association between female visitation frequency and environmental interaction, χ^2^(2, N = 50) = 13.95, *p* = 0.001 (<0.05), *Phi* = 0.52. On the other hand, the data analysis results for male visitation frequency and environmental interaction behavior do not show significant differences, indicating a weaker association between male visitation frequency and environmental interaction, χ^2^(2, N = 37) = 3.824, *p* = 0.148 (*p* > 0.05), *Phi* = 0.32.

Based on the above analysis, it can be concluded that visitation frequency is an important variable that influences the interaction between individuals and the environment. The association between visitation frequency and environmental interaction behavior is affected by gender, resulting in distinct outcomes. There is a highly significant difference between female visitation frequency and environmental interaction behavior, indicating a strong correlation between the two. On the other hand, male visitation frequency and environmental interaction do not exhibit significant differences, suggesting that they are independent of each other. This suggests that females are more inclined to engage in sensory interactions with the environment, and as their visitation frequency increases, it triggers a greater level of multi-sensory and environmental interactions. However, males tend to engage in physical interactions with the environment, regardless of the frequency, as they see coastal areas as opportunities for physical activities.

### 3.3. Therapeutic Perception Analysis

#### 3.3.1. Frequency and Therapeutic Perception

Among the 87 interviewees, in the “Seldom” interval, a total of 24 individuals perceived “Therapeutic Landscape”, 10 individuals perceived “Mental Therapeutic”, and 5 individuals perceived “Both”.

In the “Visit Every week” interval, there were 63 individuals, with 13 individuals perceiving “Therapeutic Landscape” and 13 individuals perceiving “Mental Therapeutic”, while 37 individuals perceived “Both”.

The analysis results of visitation frequency and therapeutic perception show a highly significant difference, indicating a strong correlation between visitation frequency and environmental interaction, χ^2^(2, N = 87) = 10.033, *p* = 0.007 (<0.05), *Phi* = 0.34 (Table 3).

#### 3.3.2. The Analysis of Gender and Therapeutic Perception

Among the female visitors, the number of individuals perceiving “Therapeutic Landscape” is 13, “Mental Therapeutic” is 12, and “Both” is 27. In the male group, the number of individuals perceiving “Therapeutic Landscape” is 12, “Mental Therapeutic” is 10, and “Both” is 16. The data analysis results of gender and therapeutic perception show no significant differences, indicating a weak association between gender and therapeutic perception, χ^2^(2, N = 87) = 1.750, *p* = 0.417 (>0.05), *Phi* = 0.12.

Based on this, it can be concluded that visitation frequency is an important variable that influences therapeutic perception, with a close correlation between the two. It can be inferred that the more frequent the visits, the greater the variety of perceived therapeutic types. On the other hand, gender and therapeutic perception are independent of each other, with a very weak correlation (Table 4).

## 4. Discussion

### 4.1. Interaction between Body and Environment

After the completion of the dredging and intercepting sewer systems in Xinglin Bay, the restoration of water quality has provided a waterfront area for activities. People can observe birds, enjoy sunsets, watch training sessions, and enhance their physical fitness through exercise in this area. The interaction between the body and the environment can be experienced through the boardwalk above the sea (A-7; A-27; A-34; B-12), the scent of the sea (A-43; A-55; A-59; B-7), touching the seawater (B-14), and observing migratory birds foraging along the coast (B-1; B-5; B-12; B-13).

“*Due to the favorable environment, everyone enjoys running and cycling in this area. I personally make it a point to come here for exercise every day, and it has brought about significant changes in both my body and mind. The most noticeable change is the physical transformation, as I have managed to reduce my weight from 97 kg to 70 kg*.”

(B-12)

In addition to accommodating rising tides, the waterfront platform also provides a space for people to engage in water-related activities and promotes interaction between parents and children. During the field research conducted for this study, it was frequently observed that children, accompanied by their parents, engaged in fishing and water play. The grassy square was filled with people enjoying picnics, flying kites, and camping. Parents often bring their children to the waterfront on weekends to enjoy outdoor activities and increase parent–child interaction, which helps to relax and improve their relationships.

“*The environment here is excellent and suitable for children’s activities. They enjoy playing on the grassy areas and fishing near the water’s edge. This process brings about a sense of joy and relieves the pressures of daily life and work*.”

(B-12)

Research has shown that increasing greenery not only enhances the mental well-being of residents [91] but also has positive effects on children’s cognitive function and attention [92].

“*Before coming here, I felt very gloomy, but walking around and enjoying the scenery in this place improves my mood*.”

(A-57)

Local residents engage in various daily activities along the coastline, including exercising, appreciating the beautiful scenery, relaxing, contemplating life, and visiting at different times of the day, from morning to evening.

“*I basically come out every morning to watch the sunrise, enjoy the flowers, listen to the birdsong, observe the herons leisurely fishing, and so on. In the evening, I also watch the sunset. The sunset here is famous, and many people come here specifically for it. The air has a high oxygen content, and being in this environment relaxes my entire mind and body*.”

(A-46)

Beneficial and sustained environmental changes increase the attractiveness of the environment to people. In order to relax and relieve stress, it is important to have landscape settings that incorporate natural elements, biodiversity, tranquility, and a sense of refuge [93]. However, as pointed out in the conclusion of Song, Niu [93], whether the healing green space should try to avoid social and cultural features, this study maintains a reserved attitude because this study believes that social and cultural features should be part of daily life.

### 4.2. Embodied Sense of Place

In the coastal zone’s daily activities, the interactive behaviors between the body and the environment differ significantly from those on land, thereby shaping a distinct sense of place, for example, the rowing movements while rowing on the sea (A-18; A-43; A-44; A-47; A-49) and the actions of flying and controlling a kite with the sea breeze (B-3; B-5). Rowing and flying kites are not merely physical actions but rather interactions between the body and the environment, giving rise to a sense of place.

Since the external environment is the primary source of sensory information for humans, perception of the external environment primarily occurs through human senses [94]. For instance, when rowing, one can perceive the direction of the sea currents through the tactile sensation of the oar in contact with the water. Similarly, when flying a kite, one needs to observe the direction of the sea breeze through the sense of touch, hearing, and vision.

The skyline of a city and the horizon of a coastal zone indeed have significant differences. The city skyline is primarily composed of buildings or mountains, and the line of sight moves up and down due to varying heights. However, the coastline forms a horizontal line, and the horizon of the coastal zone creates a sense of expansiveness for the interviewees (A-18; A-44; B-6).

This particular group of individuals, due to their infrequent visits, primarily rely on visual, auditory, and olfactory interactions with the environment. Their visits are often driven by nostalgia, appreciation of beautiful scenery, and the desire to relax.

“*In this context, every visit here evokes a sense of relaxation. The expansive vistas and favorable ecological conditions contribute to this sentiment, with a significant presence of egrets and various unfamiliar bird species.*”

(A-25)

Local residents, being immersed in the environment for an extended period, engage in ongoing sensory and physical interactions with their surroundings, allowing them to perceive environmental changes while experiencing physical and mental well-being. Furthermore, considering that the preferred modes of transportation for visiting the research site are bicycles and walking, it further enhances the interaction between visitors and the environment.

The interview part of this study is divided into two stages. The first interview was held on 12–16 October 2022. During this period, the closure of the epidemic in China had not ended, while the epidemic of COVID-19 lasted for nearly three years. The closure of the epidemic had trapped residents in the surrounding environment and prevented them from traveling far. People had become accustomed to looking for space to interact with the environment around them.

“*Because of the epidemic situation, we can’t run around, so we choose this place near home*.”

(B-4)

“*The university campus was closed because of the epidemic, so we couldn’t get in, so we came here*.”

(B-31)

The interview time in the second stage was 8–9 April 2023 when China had just opened after the epidemic, and the tourism enthusiasm was high. Xiamen, which was originally a popular tourist city, attracted a large number of tourists, leading to the environmental deterioration and commercialization of Xinglin Bay. The large number of tourists destroyed the original environmental characteristics of Xinglin Bay, producing a large amount of domestic garbage and breaking the original quiet living atmosphere of nearby residents, and the original feeling of quiet space away from vendors ended with the opening after the epidemic (B-21; B-24; B-31; B-36).

Place is concerned with the satisfaction of biological needs, including food, water, rest, and procedure [48]. So, a large number of residents’ samples care about water quality (n = 19; A13; A-15; A-31; A-43; A-36; A-37; A-41; A-43; A-47; A-55; A-59; B-1; B-7; B-10; B-12; B-13; B-16; B-31; B-34) and parent–child relationships (n = 19; A-14; A-29; A-41; A-48; A-51; A-53; B-3; B-5; B15; B-17, B-20; B-21; B-22; B-28; B-29; B-31; B-33; B-35; B-37). There is a negative feeling toward rest time being disrupted by tourist bustling and noise (B-21) and the environment being polluted by tourists (n = 4; B-21; B-24; B-31; B-36). The feeling toward water quality, parent–child relationships, and tranquility shape the image of home. Therefore, if there are more tourists, without the security and stability of place, the place will turn into space, and the image of home will be destroyed. Therefore, we confirm the existence of its sense of place.

### 4.3. Symbolic Landscapes

Xiamen was approved as a scenic tourism city by the central government in 2001 [95]. The coastal governance measures in Xiamen have facilitated the development of tourism landscapes. Coupled with the positioning of Xiamen as a “modern port scenic tourism city” [96], the local government emphasizes the unique regional environment and culture of southern Fujian, making the scenic features of the coastline prominent [84].

Jimei School Village is located on the southeast coast at the end of the Jimei Peninsula, near Xinglin Bay. Yet, most studies on Jimei School Village focus on the Jiageng-style architecture [97]. The architecture reflects the fusion of Chinese and Western architectural cultures showing unique architectural forms and spatial characteristics. It was designed and built by Mr. Chen Jiageng, a famous patriotic overseas Chinese leader who was born and grew up in the Jimei Peninsula. The distribution of the main buildings is concentrated in the two campuses of Jimei School Village and Xiamen University [98]. Jimei School Village, as a permanent war-free village during World War II, was lucky to retain the architecture during the rapid development of the Xiamen Special Economic Zone [86]. In 1988, Aoyuan, the tomb of Mr. Chen Jiageng, was listed as a key cultural relic protection unit by the State Council [99]. In June 2006, the Jiageng historic buildings in Xiamen University and Jimei School Village were listed as national cultural relic protection units [100]. In 2021, Xiamen City proposed a protection plan for the Jimei Mei Village historical and cultural district [101]. This plan means that the future development of the Jimei Peninsula will take Jiageng-style architecture as an important consideration.

Jiageng architecture shows Mr. Chen Jiageng’s cultural values, social behavior, and individual actions, and the historical buildings beside the coastal zone become the most concrete symbolic landscape of the Jimei Peninsula.

“*I feel that the sea plank road is very special; Environment is very clean, air is clear, and Jiageng buildings are very distinctive*.”

(A-38)

“*The water quality is good and bad, and sometimes it gives off a smell; Greening and landmark buildings can be a good background for boating*.”

(A-43)

“*The combination of the lake, vegetation, and architecture creates a unique maritime landscape*.”

(B-1)

The implementation of coastal governance measures has improved the water quality in the sea, and the released coastal zones have been developed into coastal tourism facilities in line with Xiamen’s tourism-oriented urban development [27]. The design theme of the Xinglin Bay coastal zone primarily focuses on the symbolic landscape of ecological restoration. It includes seven aspects: dredging and breach restoration, an intercepting sewer system, a boardwalk above the sea, waterfront platforms, a lawn plaza, restoration of island ecology, and landscape vegetation and resilient revetment.

The aim is to promote ecological recovery in Xinglin Bay and provide increased environmental accessibility The dredging and breach restoration project targets the original silt accumulation in Xinglin Bay, with a focus on restoring coastal ecology. In terms of landscape healing, it can regulate water levels, improve water quality, and restore biodiversity. The intercepting sewer system is dedicated to water quality restoration, creating a backup water source for Xiamen and alleviating water scarcity. Local residents living near Xinglin Bay are more likely to perceive the impact of these two landscape projects:

“*When I was a child, my brother and I used to raise ducks here. Due to pollution, the water quality remained eutrophic, and there was often a foul smell. Now, the situation has improved a lot*.”

(A-13)

“*In 2015, there used to be a frequent smell, but now it’s barely noticeable, and the water quality is gradually improving. The air, environment, and greenery here are great. I often take walks and exercise on the boardwalk above the sea, and it feels refreshing*.”

(B-13)

The boardwalk above the sea connects the water’s edge, relieving pressure on the waterfront while providing visitors with a space for water-related activities, sightseeing, and exercise. The waterfront platforms are constructed using corrosion-resistant granite materials and feature stepped levels, meeting people’s desire for water proximity while buffering the rising water levels during high tide. The lawn plaza not only offers leisure and family spaces but also contributes to ecological restoration and increased green coverage.

The establishment of a heron ecological conservation area aims to restore island ecology. Preserving the original vegetation and planting mangroves stabilizes island ecology, prevents erosion, and purifies water quality. Apart from seasonal bird migrations, Xinglin Bay also hosts various bird species on a regular basis, indicating a positive trend in ecological improvement and an increasing variety of biological species(Table 5).

“*In spring (March to May), a large number of seabird hover around this area, especially near the boardwalk above the sea. It’s a densely populated scene, even reported by China Central Television*.”

(B-7)

*There are a lot of herons, and many people come to birdwatch on weekends*.

(B-12)

**Table 5:** The therapeutic landscape of Xinglin Bay Coastal Zone.

Xinglin Bay Governance Methods	Interaction between Body and Environment	Embodied Sense of Place (Satisfaction of Biological Needs, Including Food, Water, Reproduction, Rest, Dwell, Home)	Symbolic Landscapes (Cultural Values, Social Behavior, and Individual Actions of Geographical Features)	Ecological Restoration	Psychological Therapeutic Indicators	Samples
Listing the areas of national cultural relic protection units	Being a resident and visiting frequently	**Dwelling** near the protection units (reproduction)	Jiageng-style architecture (cultural values, individual actions)	Cultural ecology of waterfront	Providing a sense of security and stability	A-38, A-43, etc.
Dredging project and ecological restoration	Rowing	Experiencing the differences in **water** quality (water)	Dredging and breach restoration (cultural values)	Regulating water levels	Broadening one’s mind	A-43, etc.
Establishing a sewage interception system	Promoting long-term physical activity	Reduced odor, facilitating **bird** observation (food)	Intercepting sewer system (cultural values)	Restoring water quality	Alleviating anxiety	B-10, etc.
Connecting the two ends of the coast with moving lines	Scenic viewing; cycling; running	**Experiencing** the changes of the sunset; observing birds; **enjoying** fresh air; improving physical fitness (rest)	Boardwalk above the sea (individual actions)	Diverting pedestrian flow to reduce the pressure on the waterfront environment	Enjoyment of body and mind, releasing stress	A-7, A-34, etc.
Differential steps, granite material sub-level space	Scenic viewing; water play; paying attention to plant aromas; overlooking the sea surface; performing some stretching exercises	Direct contact with **water** (water)	Waterfront platform (individual actions)	Buffering the rising water levels during high tide	Enjoyment and relaxation, free from anxiety	B-14, B-10, etc.
Planting a lawn	Picnicking; flying kites; rest	Observing migratory **birds** and fish in the **water**, increasing outdoor activities, **parent–child** relationship, and experiencing **water** quality (food, rest, water)	Lawn plaza (social behavior)	Restoring the ecological environment and increasing green coverage	Mental image of home. A place to provide water, reproduction, and rest	B-3, B-5, etc.
Preserving the original vegetation	Appreciating the different landscape states of plants throughout the four seasons; experiencing nature	Enjoying the fresh air and appreciating the visual, auditory, and olfactory changes of **plants** throughout the four seasons (food)	Restoration of island ecology (cultural values)	Restoring the ecological environment	Relaxed mood, reducing stress	B-10, B-11, A-46, etc.
Planting trees that absorb impurities and harmful odors						
Planting coastal mangroves	Admiring the island scenery and experiencing visual, auditory, and olfactory interactions with the island while rowing	**Bird** droppings can emit strong odors; rich landscape hierarchy (food)		Stabilizing island ecology, preventing erosion, purifying water quality, providing wind protection, and reducing the impact of storm surges and waves	Mental image of home. A place to provide food	A-44, A-49, etc.
Selecting water-resistant and pollutant-absorbing plants for waterfront areas and constructing step-like artificial natural revetments	Sightseeing and exercising	Fresh air and a **quiet** environment (rest)	Landscape vegetation and resilient revetment (cultural values)	Adapting to seasonal floods	Relieving stress, breaking free from the cycle of exhaustion	A-22, A-21, B-10, etc.

### 4.4. New Spatial Relations: A Therapeutic Landscape for Untoward Events

Because of the negative experiences caused by untoward events, residents in the study area reflect on the differences in environmental quality before and after the epidemic in the interaction between the senses, body, and the environment; people input sensations, pick up information, and construct useful functions, forming a specific new spatial relationship in the context of time and space.

This new spatial relationship is assembled from two major landscape elements: one is the water body in the coastal zone, and the other is the cultural symbol of the peninsula. The water body in the coastal zone has content that satisfies biological needs, including water, and what residents care about most is water quality. Water has a long tradition of healing powers [5]. In terms of food, migratory birds and fish that are common on the waterfront are represented. In terms of reproductive spaces, participants chose to dwell near the coastal zone, to be a resident, and to develop family relationships with others. Rest places, such as lawns for developing parent-child relationships and places that require tranquility and cleanness, like home, are the most representative

The symbolic landscapes come from Jiageng-style architecture, which is representative of cultural values and individual actions. It becomes the background of Xinglin Bay and is also a psychological symbol of local residents. Various measures to restore the ecology of the coastal zone of Xinglin Bay can be seen in order to display ecological and cultural values; the lawn plaza is a good place for the development of social behavior. The waterfront platform and the boardwalk above the sea are both main spaces for individual activities.

**Figure 5:**
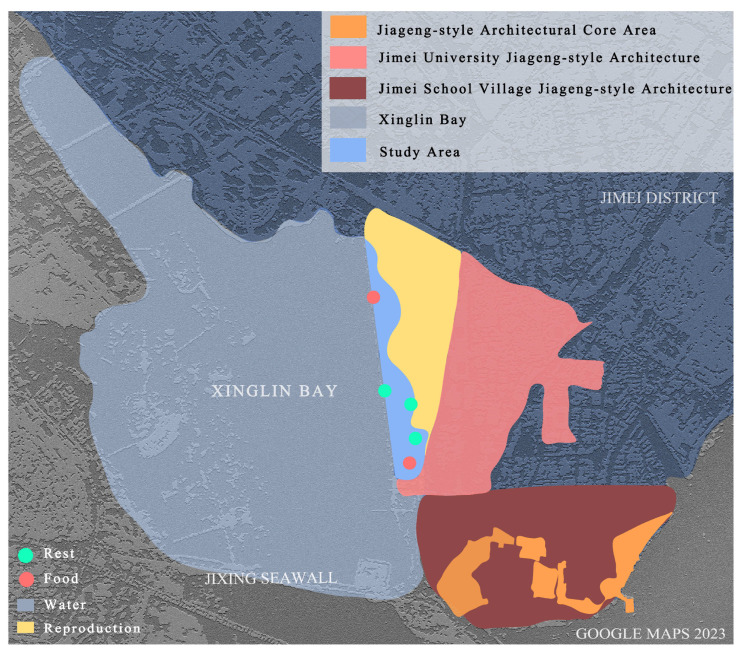
The assembly of new spatial relationships.

This new spatial relationship exists in the daily activities of residents. Before the epidemic, there were few visitors, but after the epidemic, there were more tourists. The landscape is the same, but the purpose of residents’ visits is different because of the affordances emphasized. Based on untoward event experience, a home-like feature that can satisfy biological needs and has security and stability is assembled into a new spatial relationship. (Figure 5).

## 5. Conclusions

The purpose of this article is to explore which kinds of elements of everyday life in coastal zones come together to form a therapeutic landscape and how and why. This study proposes the concept of a loop (as Figure 1) that attempts to explain the causal relationship behind the therapeutic function in the daily landscape of the coastal zone.

In the case of Xinglin Bay, the two-interview data before and after the epidemic in this study have important meaning.

The first interview was conducted during the COVID-19 epidemic that had been going on for nearly three years. We found that the long-term blockade caused negative psychological experiences, which stimulated residents’ reflection on healthy life. The memory before the epidemic and the projected mental image formed environmental perception. Because residents were stimulated by the experience, it drives more sensory input and selective attention to the coastal zone. In order to deal with the experiences, residents pick up elements that have an affordance for themselves in their daily life.

The calm water surface of the coastal zone, fish and migratory birds, parent–child activity lawns, distant Jiageng architecture, and residential buildings form new spatial relationships through conscious assembly. Through frequent visits, they immerse themselves in home-like safety and stability, which is a landscape with therapeutic functions. In the second phase of interviews, China had just opened up after the epidemic, and Xiamen attracted a large number of tourists to visit. The daily life of residents was disrupted by the negative behavior of tourists, once again forming negative experiences. Residents began to reflect on the quiet and clean memories of the coastal zone in the past. If a mental image like home is broken, environmental interactive behavior may not occur.

This study suggests that once the loop of the new spatial relationship is disrupted, the healing function in the landscape no longer exists. Furthermore, this therapeutic landscape may only be accomplished with the support of sufficient frequency. This new spatial relationship can be seen as an evolutionary model for humans to make full use of environmental functions and pursue better conditions for themselves.

## Figures and Tables

**Table 1:** Cross-analysis of visit frequency and environmental interaction.

Subject	Classification	Analysis	Behaviors Interacting with the Environment			
			Senses	Body	Both	Total
Frequency	Seldom	Count	17	1	6	24
		Expected count	8.6	5.2	10.2	24
		% within frequency	70.80%	4.20%	25.00%	100.00%
		% within behaviors interacting with the environment	54.80%	5.30%	16.20%	27.60%
		% of total	19.50%	1.10%	6.90%	27.60%
	Visit Every week	Count	14	18	31	63
		Expected count	22.4	13.8	26.8	63
		% within frequency	22.20%	28.60%	49.20%	100.00%
		% within behaviors interacting with the environment	45.20%	94.70%	83.80%	72.40%
		% of total	16.10%	20.70%	35.60%	72.40%
Total		Count	31	19	37	87
		Expected count	31	19	37	87
		% within frequency	35.60%	21.80%	42.50%	100.00%
		% within behaviors interacting with the environment	100.00%	100.00%	100.00%	100.00%
		% of total	35.60%	21.80%	42.50%	100.00%

**Table 2:** Cross-tabulation of gender–environment interaction behavior.

Subject	Classification	Analysis	Interacting with the Environment			
			Senses	Body	Both	Total
Gender	Female	Count	22	9	19	50
		Expected count	17.8	10.9	21.3	50.0
		% within gender	44.0%	18.0%	38.0%	100.0%
		% within behaviors interacting with the environment	71.0%	47.4%	51.4%	57.5%
		% of total	25.3%	10.3%	21.8%	57.5%
	Male	Count	9	10	18	37
		Expected count	13.2	8.1	15.7	37.0
		% within gender	24.3%	27.0%	48.6%	100.0%
		% within behaviors interacting with the environment	29.0%	52.6%	48.6%	42.5%
		% of total	10.3%	11.5%	20.7%	42.5%
Total		Count	31	19	37	87
		Expected count	31.0	19.0	37.0	87.0
		% within gender	35.6%	21.8%	42.5%	100.0%
		% within behaviors interacting with the environment	100.0%	100.0%	100.0%	100.0%
		% of total	35.6%	21.8%	42.5%	100.0%

**Table 3:** Cross-tabulation of visit frequency (weeks) and perception of therapeutic type.

Subject	Classification	Analysis	Therapeutic Type			
			Therapeutic Landscape	Mental Therapeutic	Both	Total
Frequency	Seldom	Count	10	9	5	24
		Expected count	6.3	6.1	11.6	24.0
		% within frequency	41.7%	37.5%	20.8%	100.0%
		% within therapeutic type	43.5%	40.9%	11.9%	27.6%
		% of total	11.5%	10.3%	5.7%	27.6%
	Visit Every week	Count	13	13	37	63
		Expected count	16.7	15.9	30.4	63.0
		% within frequency	20.6%	20.6%	58.7%	100.0%
		% within therapeutic type	56.5%	59.1%	88.1%	72.4%
		% of total	14.9%	14.9%	42.5%	72.4%
Total		Count	23	22	42	87
		Expected count	23.0	22.0	42.0	87.0
		% within frequency	26.4%	25.3%	48.3%	100.0%
		% within therapeutic type	100.0%	100.0%	100.0%	100.0%
		% of total	26.4%	25.3%	48.3%	100.0%

**Table 4:** Cross-tabulation of gender and therapeutic type.

Subject	Classification	Analysis	Therapeutic Type			
			Landscape Therapeutic	Mental Therapeutic	Both	Total
Gender	Female	Count	11	12	27	50
		Expected count	13.2	12.6	24.1	50.0
		% within gender	22.0%	24.0%	54.0%	100.0%
		% within therapeutic type	47.8%	54.5%	64.3%	57.5%
		% of total	12.6%	13.8%	31.0%	57.5%
	Male	Count	12	10	15	37
		Expected count	9.8	9.4	17.9	37.0
		% within gender	32.4%	27.0%	40.5%	100.0%
		% within therapeutic type	52.2%	45.5%	35.7%	42.5%
		% of total	13.8%	11.5%	17.2%	42.5%
Total		Count	23	22	42	87
		Expected count	23.0	22.0	42.0	87.0
		% within gender	26.4%	25.3%	48.3%	100.0%
		% within therapeutic type	100.0%	100.0%	100.0%	100.0%
		% of total	26.4%	25.3%	48.3%	100.0%

## Data Availability

The data presented in this study are available on request from the corresponding author.

## Supplementary Materials

The following supporting information can be downloaded at: https://www.mdpi.com/article/10.3390/bs13100794/s1. Table S1: Sample data and interview summary.

## References

[1] Ulrich R.S., Simons R.F., Losito B.D., Fiorito E., Miles M.A., Zeleson M. (1991). Stress recovery during exposure to natural and urban environments. J. Environ. Psychol..

[2] Ulrich R.S. (1979). Visual landscapes and psychological well-being. Landsc. Res..

[3] Ulrich R.S. (1981). Natural versus urban scenes: Some psychophysiological effects. Environ. Behav..

[4] Gidlow C.J., Jones M.V., Hurst G., Masterson D., Clark-Carter D., Tarvainen M.P., Smith G., Nieuwenhuijsen M. (2016). Where to put your best foot forward: Psycho-physiological responses to walking in natural and urban environments. J. Environ. Psychol..

[5] Gesler W.M. (1992). Therapeutic landscapes: Medical issues in light of the new cultural geography. Soc. Sci. Med..

[6] Gesler W.M. (1993). Therapeutic landscapes: Theory and a case study of Epidauros, Greece. Environ. Plan. D Soc. Space.

[7] Conradson D. (2005). Landscape, care and the relational self: Therapeutic encounters in rural England. Health Place.

[8] Bell S.L., Foley R., Houghton F., Maddrell A., Williams A.M. (2018). From therapeutic landscapes to healthy spaces, places and practices: A scoping review. Soc. Sci. Med..

[9] Bell S.L., Hickman C., Houghton F. (2023). From therapeutic landscape to therapeutic ‘sensescape’ experiences with nature? A scoping review. Wellbeing Space Soc..

[10] Carolan M.S. (2009). ‘I do therefore there is’: Enlivening socio-environmental theory. Environ. Polit..

[11] Vannini P., Ahluwalia-Lopez G., Waskul D., Gottschalk S. (2010). Performing Taste at Wine Festivals: A Somatic Layered Account of Material Culture. Qual. Inq..

[12] Jeffries J.M. (2018). Negotiating acquired spinal conditions: Recovery with/in bodily materiality and fluids. Soc. Sci. Med..

[13] Steven D.B., Paula R. (2019). Vital spaces and mental health. Med. Humanit..

[14] Yu C.-P., Lee H.-Y., Luo X.-Y. (2018). The effect of virtual reality forest and urban environments on physiological and psychological responses. Urban For. Urban Green..

[15] Zabini F., Albanese L., Becheri F., Gavazzi G., Giganti F., Giovanelli F., Gronchi G., Guazzini A., Laurino M., Li Q. (2020). Comparative Study of the Restorative Effects of Forest and Urban Videos during COVID-19 Lockdown: Intrinsic and Benchmark Values. Int. J. Environ. Res. Public Health.

[16] Mohamad Yahaya N.A., Awang Rambli D.R., Sulaiman S., Merienne F., Alyan E. (2023). Design of Game-Based Virtual Forests for Psychological Stress Therapy. Forests.

[17] Syed Abdullah S.S., Awang Rambli D.R., Sulaiman S., Alyan E., Merienne F., Diyana N. (2021). The Impact of Virtual Nature Therapy on Stress Responses: A Systematic Qualitative Review. Forests.

[18] Rajoo K.S., Karam D.S., Abdullah M.Z. (2020). The physiological and psychosocial effects of forest therapy: A systematic review. Urban For. Urban Green..

[19] Yu C.-P., Hsieh H. (2020). Beyond restorative benefits: Evaluating the effect of forest therapy on creativity. Urban For. Urban Green..

[20] Lee J., Tsunetsugu Y., Takayama N., Park B.-J., Li Q., Song C., Komatsu M., Ikei H., Tyrväinen L., Kagawa T. (2014). Influence of Forest Therapy on Cardiovascular Relaxation in Young Adults. Evid. -Based Complement. Altern. Med..

[21] Chae Y., Lee S., Jo Y., Kang S., Park S., Kang H. (2021). The Effects of Forest Therapy on Immune Function. Int. J. Environ. Res. Public Health.

[22] Lee S.-H., Chu Y.-C., Kung P.-C. (2022). Taiwan’s forest from environmental protection to well-being: The relationship between ecosystem services and health promotion. Forests.

[23] Yu C.-P., Lin C.-M., Tsai M.-J., Tsai Y.-C., Chen C.-Y. (2017). Effects of Short Forest Bathing Program on Autonomic Nervous System Activity and Mood States in Middle-Aged and Elderly Individuals. Int. J. Environ. Res. Public Health.

[24] Lee H.-J., Lin F.-H., Kuo W.-J. (2017). The neural mechanism underpinning balance calibration between action inhibition and activation initiated by reward motivation. Sci. Rep..

[25] Gavazzi G., Giovannelli F., Currò T., Mascalchi M., Viggiano M.P. (2021). Contiguity of proactive and reactive inhibitory brain areas: A cognitive model based on ALE meta-analyses. Brain Imaging Behav..

[26] Beer R.D. (2000). Dynamical approaches to cognitive science. Trends Cogn. Sci..

[27] Wang H., Zou Z., Tsai S.-C. (2022). Exploring Environmental Restoration and Psychological Healing from Perspective of Resilience: A Case Study of Xinglin Bay Landscape Belt in Xiamen, China. Int. J. Environ. Sustain. Prot..

[28] Bell S.L., Phoenix C., Lovell R., Wheeler B.W. (2015). Seeking everyday wellbeing: The coast as a therapeutic landscape. Soc. Sci. Med..

[29] Bachelard G. (1983). Water and Dreams.

[30] Bachelard G. (1969). The Poetics of Space.

[31] Game A., Metcalfe A. (2011). ‘My corner of the world’: Bachelard and Bondi Beach. Emot. Space Soc..

[32] Tuan Y.-F. (2018). Topophilia: A Study of Environmental Perception, Attitudes, and Values.

[33] Gilbert E.W. (1939). The growth of Inland and seaside health resorts in England1. Scott. Geogr. Mag..

[34] Gilbert E.W., Baumgartner H. (1965). The Holiday Industry and Seaside Towns in England and Wales. Festschrift Leopold G Scheidl zum 60 Geburtstag.

[35] Sauer C.O. (1962). Seashore-Primitive Home of Man?. Proc. Am. Philos. Soc..

[36] Ryan A. (2016). Where Land Meets Sea: Coastal Explorations of Landscape, Representation and Spatial Experience.

[37] Doody J.P. (2001). Coastal Conservation and Management: An Ecological Perspective.

[38] Charlier R.H., Chaineux M.-C.P. (2009). The healing sea: A sustainable coastal ocean resource: Thalassotherapy. J. Coast. Res..

[39] Glass-Coffin B. (1991). Discourse, daño, and healing in north coastal Peru. Med. Anthropol..

[40] Erdtsieck J.J. (2003). In the Spirit of Uganga: Inspired Healing and Healership in Tanzania.

[41] Gascon M., Zijlema W., Vert C., White M.P., Nieuwenhuijsen M.J. (2017). Outdoor blue spaces, human health and well-being: A systematic review of quantitative studies. Int. J. Hyg. Environ. Health.

[42] Kearns R., Collins D. (2012). Feeling for the coast: The place of emotion in resistance to residential development. Soc. Cult. Geogr..

[43] Jiang S. (2014). Therapeutic landscapes and healing gardens: A review of Chinese literature in relation to the studies in western countries. Front. Archit. Res..

[44] Shaw R., Weimer W., Palermo D. (1974). Ecological psychology: The consequence of a commitment to realism. Cognition and the Symbolic Processes.

[45] Campbell B. (2017). Human Evolution: An Introduction to Man’s Adaptations.

[46] Gibson J.J. (1979). The Theory of Affordances. The Ecological Approach to Visual Perception.

[47] Cambridge University (2023). Cambridge Dictionary: Cambridge University Press and Assessment 2023. https://dictionary.cambridge.org/.

[48] Tuan Y.-F. (2003). Space and Place: The Perspective of Experience.

[49] McAndrew F.T. (2020). Environmental Psychology.

[50] Ittelson W.H., Franck K.A., O’Hanlon T.J. (1976). The nature of environmental experience. Experiencing the Environment.

[51] Tran K. (2006). Public perception of development issues: Public awareness can contribute to sustainable development of a small island. Ocean. Coast. Manag..

[52] Tran K.C., Euan J., Isla M.L. (2002). Public perception of development issues: Impact of water pollution on a small coastal community. Ocean. Coast. Manag..

[53] Lin J.-Y., Liaw S.-C. (2006). Improvement and Management of Stream Corridors in the Yi-Lan River: Analysis of Environmental Perveption. J. Chin. Soil Water Conserv..

[54] Lin L.-F., Huang G.-H., Wang Y. (2010). Research on the Evaluation System of the Authenticity of Ethnic Cultural Tourism Products Based on Factor Analysis. Hum. Geogr..

[55] Gesler W. (2005). Therapeutic landscapes: An evolving theme. Health Place.

[56] Doughty K., Hu H., Smit J. (2022). Therapeutic landscapes during the COVID-19 pandemic: Increased and intensified interactions with nature. Soc. Cult. Geogr..

[57] Olszewska-Guizzo A., Fogel A., Escoffier N., Ho R. (2021). Effects of COVID-19-related stay-at-home order on neuropsychophysiological response to urban spaces: Beneficial role of exposure to nature?. J. Environ. Psychol..

[58] Jellard S., Bell S.L. (2021). A fragmented sense of home: Reconfiguring therapeutic coastal encounters in COVID-19 times. Emot. Space Soc..

[59] Meinig D.W., Meinig D.W. (1979). Introduction. The Interpretation of Ordinary Landscapes.

[60] Rowntree L.B., Conkey M.W. (1980). Symbolism and the cultural landscape. Ann. Ass. Am. Geog..

[61] Cosgrove D., Daniels S., Cosgrove D., Daniels S. (1988). Introduction: Iconography and landscape. The Iconography of Landscapes.

[62] Wagner P.L. (1975). The themes of cultural geography rethought. Yearb. Assoc. Pac. Coast Geogr..

[63] Fountoulakis K.N., Apostolidou M.K., Atsiova M.B., Filippidou A.K., Florou A.K., Gousiou D.S., Katsara A.R., Mantzari S.N., Padouva-Markoulaki M., Papatriantafyllou E.I. (2021). Self-reported changes in anxiety, depression and suicidality during the COVID-19 lockdown in Greece. J. Affect. Disord..

[64] Peteet J.R. (2020). COVID-19 anxiety. J. Relig. Health.

[65] Rehman U., Shahnawaz M.G., Khan N.H., Kharshiing K.D., Khursheed M., Gupta K., Kashyap D., Uniyal R. (2021). Depression, anxiety and stress among Indians in times of Covid-19 lockdown. Community Ment. Health J..

[66] Dettmann L.M., Adams S., Taylor G. (2022). Investigating the prevalence of anxiety and depression during the first COVID-19 lockdown in the United Kingdom: Systematic review and meta-analyses. Br. J. Clin. Psychol..

[67] Albagmi F.M., AlNujaidi H.Y., Al Shawan D.S. (2021). Anxiety levels amid the COVID-19 lockdown in Saudi Arabia. Int. J. Gen. Med..

[68] Bakker E.D., van der Pas S.L., Zwan M.D., Gillissen F., Bouwman F.H., Scheltens P., van der Flier W.M., van Maurik I.S. (2023). Steeper memory decline after COVID-19 lockdown measures. Alzheimer’s Res. Ther..

[69] Radka K., Wyeth E.H., Derrett S. (2022). A qualitative study of living through the first New Zealand COVID-19 lockdown: Affordances, positive outcomes, and reflections. Prev. Med. Rep..

[70] Gualano M.R., Lo Moro G., Voglino G., Bert F., Siliquini R. (2021). Monitoring the impact of COVID-19 pandemic on mental health: A public health challenge? Reflection on Italian data. Soc. Psychiatry Psychiatr. Epidemiol..

[71] Chirisa I., Mavhima B., Nyevera T., Chigudu A., Makochekanwa A., Matai J., Masunda T., Chandaengerwa E.K., Machingura F., Moyo S. (2021). The impact and implications of COVID-19: Reflections on the Zimbabwean society. Soc. Sci. Humanit. Open.

[72] Chaudhary M., Sodani P., Das S. (2020). Effect of COVID-19 on economy in India: Some reflections for policy and programme. J. Health Manag..

[73] Adams T., Kopelman S. (2022). Remembering COVID-19: Memory, crisis, and social media. Media Cult. Soc..

[74] Herrmann D., Oudman E., Postma A. (2023). The era of our lives: The memory of Korsakoff patients for the first COVID-19 pandemic lockdown in the Netherlands. Conscious. Cogn..

[75] Bai H. (2020). A critical reflection on environmental education during the COVID-19 pandemic. J. Philos. Educ..

[76] Mollica R.F., Fernando D.B., Augusterfer E.F. (2021). Beyond Burnout: Responding to the COVID-19 Pandemic Challenges to Self-Care. Curr. Psychiatry Rep..

[77] Wang K., Cui Q., Xu H. (2018). Desert as therapeutic space: Cultural interpretation of embodied experience in sand therapy in Xinjiang, China. Health Place.

[78] Zhou P. (2021). Affordable and enjoyable health shopping: Commodified therapeutic landscapes for older people in China’s urban open spaces. Health Place.

[79] Zhou P., Grady S.C., Rosenberg M.W. (2021). Creating therapeutic spaces for the public: Elderly exercisers as leaders in urban China. Urban Geogr..

[80] Wang Y., Ji X.-m. (2011). Environmental Characteristics and Changes of Coastal Ocean as Land-Ocean Transitional Zone of China. Sci. Geogr. Sin..

[81] Huang L.-H., Jiang Y., Lin c Li T.-W., Chen F., Wang W.-Y. (2020). Research on the coupling coordination relationship between Xiamen Port development and coastal eco-nvironment evolution. Environ. Pollut. Prev..

[82] Wang T., Hu M., Song L., Yu J., Liu R., Wang S., Wang Z., Sokolova I.M., Huang W., Wang Y. (2020). Coastal zone use influences the spatial distribution of microplastics in Hangzhou Bay, China. Environ. Pollut..

[83] Chen S., Pearson S.G. (2015). Managing China ‘ s Coastal Environment: Using a Legal and Regulatory Perspective. Int. J. Environ. Sci. Dev..

[84] Zou Z., Zhang Y.-Y., Lee S.-H., Tsai S.-C. (2023). The Transformation of Coastal Governance, from Human Ecology to Local State, in the Jimei Peninsula, Xiamen, China. Water.

[85] XiamenOceanandFisheryBureau (2018). Treading the Waves and Flying Songs: Oral Records of Key Figures in the Twenty Years of Comprehensive Management of the Coastal Zone in Xiamen from 1996 to 2016.

[86] Zhang Y.-Y., Zou Z., Tsai S.-C. (2022). From Fishing Village to Jimei School Village: Spatial Evolution of Human Ecology. Int. J. Environ. Sustain. Prot..

[87] Ying J. (2010). Xiamen: An ecological landscape belt will be built around Xinglin Bay to borrow scenery from the Garden Expo. Xiamen Daily.

[88] Qiu H.-Z. (2013). Quantitative Research and Statistical Analysis SPSS <PASW> Data Analysis Paradigm Analysis.

[89] Evans G.W., Brennan P.L., Skorpanich M.A., Held D. (1984). Cognitive mapping and elderly adults: Verbal and location memory for urban landmarks. J. Gerontol..

[90] Antes J.R., McBride R.B., Collins J.D. (1988). The effect of a new city traffic route on the cognitive maps of its residents. Environ. Behav..

[91] Kuo F.E., Sullivan W.C. (2001). Aggression and Violence in the Inner City:Effects of Environment via Mental Fatigue. Environ. Behav..

[92] Wells N.M. (2000). At Home with Nature: Effects of “Greenness” on Children’s Cognitive Functioning. Environ. Behav..

[93] Song R., Niu Q.-C., Zhu L., Gao T., Qiu L. (2018). Construction of Restorative Environment Based on Eight Perceived Sensory Dimensions in Green Spaces—A Case Study of the People’s Park in Baoji. Green Infrastruct..

[94] Durie B. (2005). Doors of perception. New Sci..

[95] State Council, China CPsGotPsRo (2000). Reply of the State Council on the Overall Urban Planning of Xiamen City.

[96] Fang Q.-H. (2006). Strategic Environmental Assessment for Ecosystem Management in Coastal Areas. Ph.D. Dissertation.

[97] Daguang W., Zhenkun L. (2021). Cultural Implications in the Names of Jiageng Buildings in Xiamen and Jimei Universities. J. Jishou Univ. (Soc. Sci. Ed.).

[98] Su H., Zhang K., Wang H. (2015). Research on Fujian Marine Culture.

[99] State Council Notice of the State Council Municipality on Announcing the Third Batch of National Key Cultural Relics Protection Units: State Council Municipality; 1988 [updated 2014/07/21]. https://www.gov.cn/guoqing/2014-07/21/content_2721163.htm.

[100] State Council (2006). Notice of the State Council on Approving and Promulgating the Sixth Batch of National Key Cultural Relics Protection Units china: State Council. https://www.gov.cn/gongbao/content/2006/content_346203.htm..

[101] Bureau XMNRaP (2021). Xiamen Jimei School Village Historical and Cultural District Protection Plan Approved: Xiamen Municipal Natural Resources and Planning Bureau. http://fj.people.com.cn/BIG5/n2/2021/1021/c181466-34967731.html.

