# Supplementary material for: Cognition and Interaction: From the Perspective of Daily Therapeutic Landscape of the Coastal Zone
Source: Behav Sci (Basel). 2023 Sep 25;13(10):794. doi: 10.3390/bs13100794 (PMC10604492; doi:10.3390/bs13100794)
Supplement: Supplementary file 1 [file behavsci-13-00794-s001.zip › behavsci-2545002-supplementary.pdf]

Table S1 Sample data and interview summary

	A	B	C	D	E	F	G	H
1	No.	Sample Coding	Gender	Frequency（per week）	Age	Way of interacting with the environment	Purpose	interview summary
2	1	A1	F	4-6 times	20-35	Both	Stress	Engaging in regular physical exercise, such as running, serves as a beneficial method for alleviating the considerable pressures of daily life.
3	2	A2	M	Seldom	Under 20	Both	Scenery	The scenery is quite pleasing.
4	3	A3	F	1-3 times	20-35	Body	Relaxation	Each time I visit, I experience a profound sense of relaxation and rejuvenation, both mentally and physically.
5	4	A4	F	4-6 times	20-35	Both	Scenery Relaxation	By unwinding and broadening one's horizons, accompanied by a romantic partner, one can relax both physically and mentally, while enjoying the breathtaking scenery.
6	5	A5	F	Everyday	46-65	Both	Scenery	The environment is favorable, and exercise is convenient.
7	6	A6	M	Everyday	46-65	Body	Scenery Health	Engaging in leisure activities and physical fitness pursuits contribute positively to one's physical well-being, while also providing the opportunity to appreciate expansive landscapes.
8	7	A7	M	Seldom	36-45	Senses	Scenery	Appreciating the sunset.
9	8	A8	M	4-6 times	Under 20	Both	Scenery Health	Enhancing physical fitness and improving mood.
10	9	A9	M	Seldom	Under 20	Body	Scenery	Contemplating the scenery.
11	10	A13	M	1-3 times	46-65	Body	Health	During my childhood, I resided in the vicinity and used to raise ducks here alone. Frequently, my siblings and I revisit this place to reminisce about our childhood memories and to perceive the passage of time. In recent years, there has been a noticeable improvement in water quality. The captivating scenery allows us to pursue a sense of belonging.
12	11	A15	M	1-3 times	20-35	Both	Scenery Relaxation	The environment and cleanliness aspects are comparatively excellent, and being in this area brings about a sense of happiness. Furthermore, the seawater quality has improved.
13	12	A16	M	4-6 times	46-65	Both	Scenery Stress	Running brings about a positive mood. The landscape features various scenic facilities such as walking trails, sunset views, and water features. It provides an opportunity to contemplate life, detach oneself from worldly concerns, and adjust one's mindset. The ecological environment is favorable, frequently observed with fish (poaching) and white herons.
14	13	A17	M	Everyday	46-65	Body	Scenery	The physique becomes leaner. The pleasant environment and fresh air in the vicinity create a sense of tranquility. White herons, mandarin ducks (rarely), and wild ducks are frequently seen. The scenery while boating is remarkable.
15	14	A19	F	Everyday	46-65	Senses	Scenery	Observing the sunset and the people rowing boats evokes a sense of tranquility and uplifts the mood.
16	15	A20	M	4-6 times	20-35	Both	Relaxation	Being in this place leads to an improvement in mood.
17	16	A21	F	4-6 times	36-45	Both	Scenery Relaxation	The air quality is excellent. The sunset exhibits rich variations. If I haven't been here for several days, I feel discomfort in my lower back and a decline in mood. During summer, there is an abundance of peaches and waxberries. White herons are frequently observed, as there is a nearby habitat for them.
18	17	A22	F	4-6 times	46-65	Both	Scenery Health	After exercising, one feels very comfortable. With more than half a year of consistent exercise, there is a noticeable improvement in physical fitness. The scenery is remarkable, combining both artificial and natural elements, such as walking trails, vibrant evening skies, and waterfront views. It provides an opportunity to observe the changing hues of the sunset. White herons are frequently seen in groups at this location.
19	18	A23	M	4-6 times	20-35	Both	Scenery Health	Engaging in physical exercise promotes health maintenance. If the sunset is exceptionally beautiful, one would pause and appreciate the mesmerizing view.
20	19	A24	F	1-3 times	46-65	Body	Scenery Health	The scenery is impressive. Being at home can be oppressive, but stepping outside and feeling the sea breeze brings about a sense of relief and tranquility. There are numerous white herons in the area.
21	20	A25	F	Seldom	20-35	Senses	Scenery Relaxation	I feel more relaxed, in a better mood, and my perspective broadens. The aerial footage of the boardwalk captured by a drone is truly breathtaking. There are abundant white herons, indicating a thriving ecological environment.
22	21	A26	M	Everyday	20-35	Both	Scenery	There is a relatively large population of white herons. The proximity to my home provides a convenient location for leisurely walks. The waterfront is exceptionally beautiful.

Table S1 Sample data and interview summary

	A	B	C	D	E	F	G	H
1	No.	Sample Coding	Gender	Frequency（per week）	Age	Way of interacting with the environment	Purpose	interview summary
23	22	A27	M	Everyday	Over 66	Both	Health	Taking inspiration from athletes, one can exercise on the seaside boardwalk. By maintaining a regular exercise routine, one can achieve good physical health.
24	23	A28	F	Seldom	20-35	Senses	Relaxation	There is a noticeable improvement in mood, and there is an urge to approach areas with abundant vegetation and scenic landscapes.
25	24	A29	F	1-3 times	36-45	Both	Scenery Health	It is conveniently close to home and provides a place for physical exercise. Observing the illuminated lights at night was a delight, but since energy-saving measures were implemented, the brilliance of the lights has diminished. It is suitable for family activities, allowing children to play and engage in sports. It also serves as a seasonal migration route for migratory birds, and there is a relatively large population of white herons.
26	25	A30	F	1-3 times	46-65	Both	Relaxation Health	The air quality is excellent, with the refreshing sea breeze. Taking a moment to feel the wind uplifts the mood. There are wild ducks, small and adorable, indicating a thriving ecological environment.
27	26	A31	M	1-3 times	20-35	Body	Scenery Health	In the past, the seawater in this area had a strong odor during the summer. However, there has been an improvement in water quality.
28	27	A32	F	Everyday	46-65	Body	Scenery Health	The air is clear, and the scenery is beautiful. Taking a walk helps maintain health and improves mood. There is an abundance of fish in the area.
29	28	A33	M	Everyday	20-35	Both	Scenery Health	The nighttime scenery is particularly captivating, but the sunset over the water is the most alluring view for me, accompanied by the illuminated buildings on the opposite side. Exercise helps alleviate stress, and running with a group allows for conversations and social interactions. However, I prefer running alone, as it provides a rewarding experience in sync with the beautiful surroundings. Occasionally, bird sightings add to the sense of a thriving ecological environment.
30	29	A34	F	Seldom	20-35	Both	Scenery	The water cypress boardwalk is particularly unique, and it gives the impression of slightly clearer water.
31	30	A35	F	Everyday	46-65	Body	Scenery	After running, a sense of comfort emerges; the air is clear, distant from the thoroughfare; witnessing fish leaping out of the water.
32	31	A36	F	Everyday	20-35	Body	Scenery	The scenery (green plants and water) and air are both excellent; cycling improves physical fitness, enjoying the sea breeze enhances one's mood; the water quality has improved, reducing the fishy smell. Previously, due to the strong odor of the water, I chose to buy a house elsewhere.
33	32	A37	M	1-3 times	20-35	Body	Scenery Health	The nearby scenery is quite pleasing; running helps maintain good health; I have noticed an improvement in water quality recently, with a reduction in the fishy smell.
34	33	A39	M	Seldom	20-35	Senses	Relaxation	I haven't noticed any significant changes; the coastal boardwalk is still quite pleasant.
35	34	A40	F	Seldom	46-65	Both	Scenery Health	The abundance of scenery is quite noticeable; it provides opportunities for physical fitness and strengthening of the body.
36	35	A41	F	Everyday	36-45	Body	Health	Engaging in physical activity promotes good health, and simultaneously listening to audiobooks enhances both physical and mental well-being, leading to a great sense of self-fulfillment. However, I hope the water quality improves further as occasionally there are unpleasant smells emanating from it.
37	36	A42	M	1-3 times	36-45	Body	Scenery	The location is secure, and the air quality is relatively good. The overall environment is excellent. During autumn, there will be migratory birds passing through.
38	37	A43	F	4-6 times	46-65	Body	Scenery Health Relaxation	The water quality fluctuates, sometimes emitting odors. The greenery and iconic buildings serve as a great backdrop for rowing. The openness of the water creates a sense of expansiveness. There is a significant presence of migratory birds, especially during autumn.
39	38	A44	F	Everyday	46-65	Senses	Scenery	After working here for over 20 years, I have experienced visual fatigue. Every day, there is the migration of migratory birds, with egrets and cormorants scattered around. The ecological environment feels quite good, with numerous bird-inhabited islands in the water. When in close proximity, one can detect the smell of bird droppings.
40	39	A45	M	1-3 times	46-65	Body	Scenery Health	The overall environment is excellent, whether it is man-made or natural. Running in this area gives a sense of relaxation. However, there seems to be a limited variety of wildlife, primarily consisting of birds.

Table S1 Sample data and interview summary

	A	B	C	D	E	F	G	H
1	No.	Sample Coding	Gender	Frequency（per week）	Age	Way of interacting with the environment	Purpose	interview summary
41	40	A46	F	Everyday	Over 66	Both	Scenery Relaxation	In the morning, one can enjoy watching the sunrise, admiring flowers, listening to the birdsong, and observing egrets leisurely fishing, among other things. In the evening, there is the beauty of the sunset, with many people capturing photos of the setting sun and fiery clouds. It brings a sense of relaxation, and the air has a high oxygen content. Recently, the plants have been growing exceptionally well, with the vibrant and glossy texture of the beautiful Kapok flowers resembling plastic, filling the trees with blossoms. During the autumn and winter seasons, it is difficult to describe the multitude of birds adequately, as the spectacle is truly magnificent. The cormorants are numerous, creating a dense and breathtaking sight.
42	41	A47	F	1-3 times	46-65	Both	Scenery	The natural and man-made landscapes are incredibly beautiful. The greenery has been well-maintained, and the overall landscaping is impressive. The water quality may be slightly subpar. During autumn and winter, thousands of migratory birds fly in, creating a particularly spectacular sight. Watching athletes rowing boats allows us to appreciate their dedication and hard work.
43	42	A48	F	Everyday	46-65	Senses	Scenery Relaxation	It is a great place for parent-child activities, with good air quality and beautiful scenery. There is a sense of gracefulness in the way the egrets interact with people.
44	43	A49	M	Everyday	Under 20	Body	Relaxation	The feeling on the water is very comfortable and pleasant, as if being in one's element. The numerous bird-inhabited islands in the water add to the charm of the environment.
45	44	A50	M	Seldom	20-35	Both	Scenery Relaxation	Taking a walk and enjoying the scenery, the environment here is comparatively pleasant. After walking around, there is a sense of relaxation and joy in the body. The only minor drawback is that it can get a bit crowded at times.
46	45	A51	F	Seldom	20-35	Senses	Relaxation	Accompanying my mother for a walk and spending time with family while strolling has improved my physical and mental well-being. It's a common sight to see people fishing in the area.
47	46	A53	F	Seldom	20-35	Both	Scenery Relaxation	Bringing children to play and letting them have fun is a great way to unwind. The environment here is relaxing, and the overall surroundings are pleasant. The bicycle lanes are particularly attractive and appealing to explore.
48	47	A54	F	1-3 times	46-65	Both	Health	Engaging in physical activities is suitable in this area due to the good air quality and favorable environment. There have been significant improvements in water quality and other landscape facilities, with the addition of convenient tourist attractions, allowing residents and visitors to enjoy the scenery. It feels like my body is becoming healthier, and taking a walk outside uplifts my mood. It is common to exercise with companions in this area.
49	48	A55	F	1-3 times	46-65	Senses	Relaxation	The scenery is beautiful, but the water quality can sometimes deteriorate, resulting in a fishy smell. Living nearby, it's nice to take a walk and enjoy the surroundings.
50	49	A56	F	Seldom	20-35	Senses	Relaxation	Taking a look at the scenery, it would be even better if the sun is shining. Feel free to come and take a leisurely stroll.
51	50	A57	F	Seldom	20-35	Senses	Scenery Relaxation	Observing the scenery and capturing moments has become a popular activity. Before coming here, you felt quite down, but taking a walk in this area has noticeably improved your mood.
52	51	A59	F	1-3 times	36-45	Senses	Scenery Relaxation	Engaging in relaxation and leisure activities, taking a walk here brings about a more joyful mood. The scenery is quite beautiful, making it suitable for wellness purposes. Aside from the subpar water quality, the other landscapes are excellent. Particularly noteworthy is the coastal boardwalk, which serves multiple purposes and is perfect for walking, exercising, and sightseeing. It is a versatile and multi-functional pathway.
53	52	A60	F	Seldom	46-65	Senses	Scenery Relaxation	If the distance from my home to this area is not too far, I would love to come here every day. Today, I came with a friend to relax and unwind. Your mood has significantly improved, and being in this place feels like a healing experience.

Table S1 Sample data and interview summary

	A	B	C	D	E	F	G	H
1	No.	Sample Coding	Gender	Frequency（per week）	Age	Way of interacting with the environment	Purpose	interview summary
54	53	B1	F	1-3 times	36-45	Both	Scenery	The coastal boardwalk is wonderful, with beautiful scenery. Having breakfast here brings about a delightful mood. The combination of the lake, vegetation, and architecture creates a unique maritime landscape. By consistently running, your physical fitness has improved significantly. Previously, you often experienced respiratory inflammation, but now your body has improved significantly. During the autumn and winter seasons, there is a migration of migratory birds. The water quality has improved, with a significant reduction in odor. You have also discovered the presence of a sewage treatment system.
55	54	B2	F	Seldom	20-35	Senses	Scenery	The proximity of the home to this location is relatively close. The presence of wild waterfowl indicates a relatively healthy ecological environment.
56	55	B3	F	Seldom	36-45	Senses	Scenery	The proximity of the home to this location is comparatively close. The environment is favorable, with both artificial and natural landscapes being highly attractive. Children can engage in outdoor activities, fostering family conversations and enhancing interpersonal relationships.
57	56	B4	F	1-3 times	20-35	Senses	Scenery Relaxation	The sea and sunset are particularly captivating. Having a picnic in this area can provide relaxation and a sense of ease. There are numerous white egrets present. Regularly visiting this place to enjoy tea creates a pleasant and contented feeling. Due to the pandemic restrictions, it is necessary to stay close to home and therefore choose this nearby location. It is a wonderful place for unwinding, and having a picnic here evokes a sense of dining and enjoying tea together with family, creating a feeling of belonging. Additionally, ordering takeout is convenient in this area. However, the water quality is not optimal, despite some improvements compared to the past.
58	57	B5	F	1-3 times	36-45	Both	Scenery	The scenery and sunset are highly appealing to me. There are numerous white egrets in this area, and during certain seasons, there is an abundance of migratory birds. I have heard that snakes exist here, although I have not personally encountered any. The water quality is not satisfactory. Engaging in outdoor picnics here feels more relaxing, allowing children to play without the same level of noise and commotion as at home. This place provides us with a space for family activities, where my father and I can simply sit while the children entertain themselves. In autumn, when the weather is clear and the air quality is good, it is suitable for a family outing.
59	58	B6	M	Everyday	36-45	Both	Scenery Relaxation	In the morning, I go for a walk to breathe fresh air, dispel the haze in my body, and maintain a positive mood before heading to work. In the evening, I return to the seaside boardwalk to go for a run, aiming for a state of good health. Looking out from the coastline, the natural scenery seamlessly blends with the man-made features such as the boardwalk, buildings, and waterfront platforms. The sea and sky appear to merge into one, instantly uplifting my spirits. There are often many birds on the water, although I am not familiar with their specific species.
60	59	B7	F	Everyday	46-65	Both	Scenery Stress	When the weather is clear, the water has no noticeable odor. However, during unfavorable weather conditions, there is a strong odor. Special measures have been taken to address pollution, and compared to before, the water quality in this area has shown signs of improvement. However, the water quality in the villa area of the residential complex still needs to be addressed. Taking regular walks and soaking up the sun promotes good health and, with persistence, enhances one's mood, leading to a sense of pleasure. It is advisable for the elderly to come out for walks to release stress when they have spare time. During spring (March to May), a large number of seabirds hover around this area, particularly near the seaside boardwalk. They form a dense flock, and even China Central Television has reported on this phenomenon. The ecological conditions seem favorable, but water quality still requires improvement.
61	60	B8	F	Everyday	Over 66	Body	Scenery Health	The air quality is good, but I often experience bodily pain. Going for a walk outside can help alleviate the pain.

Table S1 Sample data and interview summary

	A	B	C	D	E	F	G	H
1	No.	Sample Coding	Gender	Frequency（ per week）	Age	Way of interacting with the environment	Purpose	interview summary
62	61	B9	F	Everyday	Over 66	Body	Scenery Relaxation	The air quality is good, and the combination of artificial and natural landscapes creates a harmonious and pleasant atmosphere. Regular physical exercise contributes to a comfortable and relaxed state. Additionally, there are also many white egrets in this area.
63	62	B10	M	4-6 times	36-45	Both	Scenery Stress	The waterfront landscape here feels like a backyard, with beautiful scenery and fresh air. From an overall perspective, the lake surface serves as the central focal point, with other elements complementing it. Coming here regularly, I often engage in stretching exercises, which help relax my body. In terms of mental well-being, the scenery helps alleviate anxiety and allows for a relaxed mindset, providing temporary relief from the pressures of an inward-focused world. Humans have not yet reached a point where they can solely rely on internal means to alleviate emotions, hence the need for external factors, such as beautiful landscapes. During nighttime, the lighting plays a significant role, and whether the lights of the buildings across the water are on or off can have an impact on one's mood. It is common to see pairs of mandarin ducks and white egrets in this area. The water quality was quite unpleasant a couple of years ago, but there has been significant improvement in recent years. When it comes to plants, I pay more attention to their fragrance, often overlooking their appearance. I particularly enjoy the scent of osmanthus flowers.
64	63	B11	F	Everyday	36-45	Both	Scenery Relaxation	Appreciating flowers, such as bougainvillea, and admiring their various colors brings long-lasting joy to my mood. White egrets can often be seen in the area, and there is an abundance of diverse fish species.
65	64	B12	M	Everyday	46-65	Both	Scenery Health	The environment is excellent, and everyone enjoys coming here for activities like cycling along the seaside boardwalk. On weekends, many people come to the boardwalk to take photos. Adopting such a lifestyle has brought about significant changes. Physically, I have gone from weighing 97kg to 70kg. When I first moved here, the environment was quite dirty, but it has significantly improved since then, with dedicated personnel responsible for cleaning and maintenance. A few years ago, the sewage treatment system was overloaded, resulting in foul odors, but now a new sewage system has been constructed, and the water quality has noticeably improved compared to previous years. This area used to be reclaimed land for agriculture, but it has since been restored to a unique scene where half is seawater and half is fresh water. There are many birdwatchers on weekends, and the variety of bird species is abundant. As I have a keen interest in plants, I have noticed the abundance of plants such as wax apples, longan, mangoes, and lychees, which bear fruit twice a year.
66	65	B13	M	Everyday	46-65	Both	Scenery Relaxation	The air quality is good, and the environment is pleasant. The combination of water scenery and the boardwalk is well-executed, with lush green vegetation. I often walk along the seaside boardwalk to exercise my body. There are an abundance of white egrets, especially during the autumn and winter seasons when there are also numerous cormorants and various migratory birds that gather here, creating a dense spectacle. Taking a leisurely stroll in this environment feels refreshing and invigorating, benefiting both the mind and body. The scenery is beautiful. In 2015, there used to be occasional unpleasant odors, but now they are no longer noticeable, and the water quality has been gradually improving.
67	66	B14	M	Everyday	20-35	Both	Scenery Stress	The scenery has a great appeal and is a significant factor in choosing to settle in this area. After a tiring day at work, taking a walk here brings about a pleasant feeling both physically and mentally.
68	67	B15	M	1-3 times	36-45	Senses	Relaxation	Spending leisure time with children and engaging in playful activities together brings a sense of relaxation.
69	68	B16	F	Seldom	46-65	Senses	Scenery Relaxation	Observing the scenery can significantly improve one's mood. The environment in Xinglin Bay is gradually improving, including the water quality, which is slowly getting better.

Table S1 Sample data and interview summary

	A	B	C	D	E	F	G	H
1	No.	Sample Coding	Gender	Frequency（per week）	Age	Way of interacting with the environment	Purpose	interview summary
70	69	B17	M	Everyday	46-65	Senses	Scenery Relaxation	The environment is excellent, combining both dynamic and serene elements. The green grassy areas provide ample space for children to run and play. This place used to be quite desolate, but after government improvements, the environment has undergone significant changes. Additionally, a new sports stadium has been built here, providing convenience for people to engage in sports activities.
71	70	B18	F	4-6 times	46-65	Both	Scenery Health Relaxation	The seawater here is beautiful, and the environment is excellent. Although there are many tall buildings that can feel a bit oppressive, the nighttime scenery is stunning. The influence of the surrounding cultural and educational district adds a unique flavor, with lakes and water bodies creating a different atmosphere. It is convenient to exercise here, and I feel my body getting better and better. Immersing oneself in the beauty of the setting sun, there is ample space for others to set up tents, providing a place for communion with nature. The surroundings are very peaceful, and there are diverse sports activities available. For instance, in the case of kayaking, both professional and amateur athletes gather here.
72	71	B19	M	1-3 times	46-65	Both	Scenery Health Relaxation	Running or participating in a half-marathon on the seaside boardwalk is a great experience. The environment is quite pleasant, and I personally enjoy the air quality and the cultural aspects of the area. The entire route of 23 kilometers is suitable for a half-marathon. In the past, this place used to be a breeding ground and was quite messy. However, compared to before, there have been significant improvements. I served as a soldier here in 1987, and at that time, the environment was dirty and chaotic. Since 2006-2007, there have been noticeable changes, with the construction of reclaimed land and improvements in the overall living environment. There is no doubt that the quality of life has been improving.
73	72	B20	M	Seldom	46-65	Both	Scenery Health	Bringing your children along and going for a run yourself in such a pleasant and comfortable environment is a great experience. It not only allows you to enjoy the surroundings but also contributes to a healthier lifestyle. The greenery and seaside views are both remarkable, adding to the overall appeal of the area.
74	73	B21	M	Everyday	20-35	Both	Scenery Health Relaxation	Spending time with your children and exercising in this area is a great way to enjoy their company while taking care of your own health. Initially, one of the appealing factors was the tranquility of the place. However, recently, there has been an increase in the number of people, which has unfortunately led to more littering. The grassy areas are quite lush, and now it has become livelier with many organized camping activities. After picnics, there tends to be a significant amount of trash left behind. Many tourists visit the seaside boardwalk to enjoy the scenery.
75	74	B22	M	4-6 times	36-45	Senses	Scenery Relaxation	Taking the children for a stroll in this area offers a combination of beautiful landscapes, lush greenery, and convenient facilities. It provides a great opportunity to spend time with your children while also taking breaks and resting. Going for a walk and being surrounded by the pleasant scenery can have a positive impact on your mood and overall well-being.
76	75	B23	F	Seldom	20-35	Senses	Scenery Relaxation	Having a picnic, playing with children, and watching the sunset in this area offer a beautiful landscape that helps to relax both the mind and body. The overall scenery is quite stunning and provides a serene environment for enjoying quality time with your children. The combination of outdoor activities, natural beauty, and the calming effect of the sunset can contribute to a sense of peace and relaxation, allowing you to unwind and recharge.
77	76	B24	F	1-3 times	36-45	Senses	Relaxation	The grassy areas in this location provide a peaceful environment away from street vendors. The surrounding atmosphere is generally tranquil, which contributes to a sense of contentment and peace of mind. However, as the number of visitors has increased over time, there is a corresponding increase in the amount of litter. Despite this, it is still possible to enjoy sightings of fish, ducklings, and pet dogs, which add to the overall charm of the area.

Table S1 Sample data and interview summary

	A	B	C	D	E	F	G	H
1	No.	Sample Coding	Gender	Frequency（ per week）	Age	Way of interacting with the environment	Purpose	interview summary
78	77	B25	F	Seldom	20-35	Senses	Scenery Relaxation	Due to proximity, I decided to take a leisurely stroll in this vicinity, as the environment here is favorable and cheerful, thereby appealing to children who enjoy playing in the area.
79	78	B28	F	Seldom	20-35	Senses	Scenery Stress	The pleasant environment in this area makes it suitable for accompanying young children, fostering a sense of well-being and alleviating the pressures of daily life and work. Personally, I find the presence of bicycle lanes particularly appealing, while children tend to enjoy the presence of grassy areas and the opportunity to engage in fishing activities near the water's edge.
80	79	B29	M	Seldom	20-35	Senses	Relaxation	On weekends, bringing children here for relaxation is highly suitable, as this area is conducive to recreational activities for children. The limited vehicular traffic creates a safe environment that is ideal for children to freely run and play.
81	80	B30	F	Seldom	20-35	Senses	Relaxation	Residing in the vicinity, I simply come here to enjoy some leisure time and unwind. However, it is worth noting that the water quality in this area occasionally exhibits an unpleasant odor.
82	81	B31	F	1-3 times	36-45	Both	Scenery Health	Engaging in outdoor activities such as walking and running is a popular choice for me. The environment here is more spacious, with grassy lawns and such. However, it is worth mentioning that the water quality in this area is relatively poor. My son also enjoys coming here to ride his bike, primarily because of the large grassy areas where he can bask in the sunlight and feel uplifted. It seems that middle-aged individuals also appreciate coming out for exercise, as a leisurely stroll in this area improves mood. Additionally, there are basketball courts available for recreational use. This area is rich in bird and fish populations. Today, the water levels are high, and there are children fishing along the banks.
83	82	B32	M	Seldom	20-35	Senses	Relaxation	The surrounding environment is quite pleasant, making it a great place to come and unwind. Many people come here to set up tents and relax. Today, there is a strong wind blowing, but when the weather is nice, the scenery is quite enjoyable, especially during the beautiful sunset.
84	83	B33	M	1-3 times	36-45	Body	Relaxation	Spending time playing with children and utilizing the weekend to adjust one's mood is a great way to relax. However, it seems that there haven't been any significant changes to the landscape facilities.
85	84	B34	M	Everyday	Over 66	Senses	Relaxation	It appears that there has been a significant change in the environment. The water quality and condition of the grassy areas have improved, which is a positive development. It's remarkable to see that there was nothing here before and now it has become a place where you can play and enjoy daily.
86	85	B35	F	Seldom	36-45	Senses	Relaxation	Bringing children here to enjoy the fresh air and providing them with two hours of outdoor time is a great way to let them connect with nature. This place offers a peaceful atmosphere with green lawns, a nearby sea, and trees, providing a natural setting for children. It's worth noting that there is a significant population of white herons in this area.
87	86	B36	F	Everyday	20-35	Senses	Scenery Stress	The sunset is aesthetically pleasing, attracting individuals seeking stress relief to this location. Previously characterized by sparse human presence, the area has now experienced an increase in litter accumulation due to a rise in camping enthusiasts. Consequently, the environment has deteriorated, and there has been an introduction of commercial elements, such as cycling activities.
88	87	B37	M	1-3 times	46-65	Senses	Scenery Relaxation	Bringing children here to play, appreciate the scenery, and observe birds is a delightful experience. Previously, there were numerous water spray facilities on the green spaces, which were not very convenient. However, they have now been removed, allowing for easier recreational activities and the option to set up tents. The situation here is reminiscent of the dragon boat pond. Going out for a walk improves one's mood, and regular walks contribute to good physical health. The air quality in this area is relatively good, and it is home to a significant population of white herons. There are also many large, black birds that frequently visit, and it's fascinating to observe their fishing behaviors.
89	Remarks: The sample coding method is: **A**, indicating that the interview site is located on the boardwalk above sea; **B**, indicating that the interview site is located on the East coast							
